# Novel lncRNA‐prader willi/angelman region RNA, SNRPN neighbour (*PWARSN)* aggravates tubular epithelial cell pyroptosis by regulating TXNIP via dual way in diabetic kidney disease

**DOI:** 10.1111/cpr.13349

**Published:** 2022-10-31

**Authors:** Yi Song, Feng Guo, Yan‐yan Zhao, Xiao‐jun Ma, Li‐na Wu, Ji‐feng Yu, Hong‐fei Ji, Ming‐wei Shao, Feng‐juan Huang, Lin Zhao, Xun‐jie Fan, Ya‐nan Xu, Qing‐zhu Wang, Gui‐jun Qin

**Affiliations:** ^1^ Department of Endocrinology and Metabolism The First Affiliated Hospital of Zhengzhou University Zhengzhou China; ^2^ Academy of Medical Sciences Zhengzhou University Zhengzhou China; ^3^ Institute of Clinical Medicine The First Affiliated Hospital of Zhengzhou university Zhengzhou China; ^4^ Department of Hematology The First Affiliated Hospital of Zhengzhou University Zhengzhou China; ^5^ Department of Nuclear Medicine The First Affiliated Hospital of Zhengzhou University Zhengzhou China

## Abstract

**Objectives:**

Elevated thioredoxin‐interacting protein (TXNIP)‐induced pyroptosis contributes to the pathology of diabetic kidney disease (DKD). However, the molecular mechanisms in dysregulated TXNIP in DKD remain largely unclear.

**Materials and methods:**

Transcriptomic analysis identified a novel long noncoding RNA—Prader Willi/Angelman region RNA, SNRPN neighbour (*PWARSN*)—which was highly expressed in a proximal tubular epithelial cell (PTEC) under high glucose conditions. We focused on revealing the functions of *PWARSN* in regulating TXNIP‐mediated pyroptosis in PTECs by targeting *PWARSN* expression via lentivirus‐mediated overexpression and CRISPR‐Cas9‐based knockout *in vitro* and overexpressing *PWARSN* in the renal cortex by AAV‐9 targeted injection *in vivo*. A number of molecular techniques disclosed the mechanisms of *PWARSN* in regulating TXNIP induced‐pyroptosis in DKD.

**Results:**

TXNIP‐NOD‐like receptor thermal protein domain associated protein 3 (NLRP3) inflammasome and PTEC pyroptosis were activated in the renal tubules of patients with DKD and in diabetic mice. Then we explored that *PWARSN* enhanced TXNIP‐driven PTECs pyroptosis *in vitro* and *in vivo*. Mechanistically, cytoplasmic *PWARSN* sponged miR‐372‐3p to promote TXNIP expression. Moreover, nuclear *PWARSN* interacted and facilitated RNA binding motif protein X‐linked (RBMX) degradation through ubiquitination, resulting in the initiation of *TXNIP* transcription by reducing H3K9me3‐enrichment at the *TXNIP* promoter. Further analysis indicated that *PWARSN* might be a potential biomarker for DKD.

**Conclusions:**

These findings illustrate distinct dual molecular mechanisms for *PWARSN*‐modulated TXNIP and PTECs pyroptosis in DKD, presenting *PWARSN* as a promising therapeutic target for DKD.

## Graphical abstract

Pyroptosis mediated by thioredoxin‐interacting protein (TXNIP) aggravates diabetic kidney disease (DKD), thus clarifying the key regulator of TXNIP‐induced cell pyroptosis may delay DKD progression. We unveiled a new long non‐coding RNA, Prader Willi/Angelman region RNA, SNRPN neighbour (*PWARSN)*, which is especially expressed in proximal tubular epithelial cell (PTEC), plasma and urinary sediments samples from patients with DKD and regulated TXNIP‐induced PTECs pyroptosis in DKD. Mechanistically, *PWARSN* regulates TXNIP expression via a dual pathway: both by sponging cytoplasmic miR‐372‐3p and by interacting with nuclear RNA binding motif protein X‐linked (RBMX). Our findings suggest that *PWARSN* can be used as a biomarker and may be a potential target for DKD diagnosis and treatment.

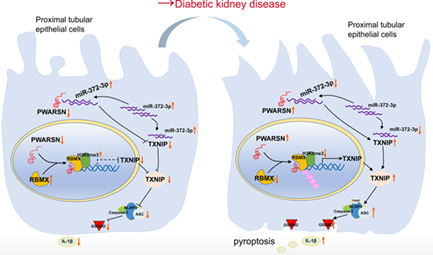

## 1 INTRODUCTION

Diabetic kidney disease (DKD) is the leading cause of end‐stage renal disease worldwide.^1^, ^2^ Previous studies have focused on the pathological injury of the glomerulus; however, multiple studies have confirmed that tubule injury plays a key role in the progression of DKD.^3^, ^4^ Metabolic disorders, changes in hemodynamics, and oxidative stress in the diabetic environment can contribute to tubular inflammation, proximal tubular epithelial cell (PTEC) death and interstitial fibrosis, which eventually progress to DKD.^5^, ^6^, ^7^ However, the pathological mechanisms that initiate and mediate tubular epithelial injury in DKD remain poorly understood.

Pyroptosis is a type of programmed cell death induced by sterile inflammation and is driven by pore formation by Gasdermin D within the plasma membrane with consequent rapid plasma membrane rupture and release of intracellular contents and proinflammatory mediators.^8^, ^9^ In the presence of excessive accumulation of reactive oxygen species (ROS), NOD‐like receptor thermal protein domain associated protein 3 (NLRP3) recruits nucleation of apoptosis‐related speckle‐like protein (ASC) and activates caspase‐1, subsequently causing cell pyroptosis.^10^ Current evidence reports on the stimulatory role of ROS in renal cell pyroptosis, and different signaling pathways have been shown to regulate pyroptosis initiation, aggravating a tubular injury, glomerular sclerosis, and renal fibrosis in DKD.^11^, ^12^, ^13^

Thioredoxin‐interacting protein (TXNIP) belongs to the thioredoxin system and binds with thioredoxin, which can resist oxidative stress in resting cells.^14^ Many studies have reported that hyperglycemia promotes the separation of TXNIP from thioredoxin, resulting in excessive oxidative stress and ROS production.^15^ Previous studies by our laboratory and others have reported that TXNIP plays a key role in the progression of DKD.^16^ Moreover, other studies have revealed that TXNIP‐induced ROS accumulation initiates pyroptosis in an NLRP3 inflammasome‐dependent manner in DKD.^17^ However, little is known about the function of TXNIP in PTEC pyroptosis and the underlying molecular mechanisms involved in the dysregulation of TXNIP in PTECs in DKD.

Long noncoding RNAs (lncRNAs) can be pivotal gene regulators by interacting with RNA, DNA and RNA‐binding proteins in different cellular processes.^18^ LncRNAs play a vital role in various physiological and pathological conditions including cell differentiation, metabolism and cancer progression.^19^, ^20^, ^21^ Emerging evidence suggests that lncRNAs are involved in the regulation of DKD.^22^ However, the influence of lncRNAs on renal tubule injury in DKD remains largely unknown. Here, we demonstrate PTEC pyroptosis in both experimental models of DKD and in patients with DKD. We show that renal tubular inflammation‐induced pyroptosis positively correlates with the elevation of a novel lncRNA—Prader Willi/Angelman region RNA, SNRPN neighbour (*PWARSN*)—which induces aberrant TXNIP expression in both the cytoplasm and nucleus via a dual pathway. Collectively, lnc*‐PWARSN* is a key modulator of TXNIP, linking pyroptosis to the progression of DKD, and may serve as a biomarker of DKD.

## 2 METHODS AND MATERIALS

### 2.1 Ethics

The study procedures involving patients were approved by the ethical committee of the First Affiliated Hospital of Zhengzhou University (Approval number: 2019‐KY‐228) and based on the ethical principles of the Helsinki Declaration.

The study procedures involving animals were conducted in accordance with the Principles of Laboratory Animal Care, and were approved by the institutional committee of the Animal Research Committee and Animal Ethics Committee of Henan Key Laboratory for Pharmacology of liver diseases (Approval number: 2019‐44) and based on the ethical principles of the Helsinki Declaration.

### 2.2 Cell lines and cell culture

Human proximal tubular epithelial cells (HK‐2 cells), mouse renal tubular epithelial cells (mRTEC), human podocyte (HPC), and human mesangial cell were purchased from the Chinese Academy of Sciences (Shanghai, China) and were cultured in Dulbecco's modified Eagle's medium (Hyclone, UT) containing 10% heat‐inactivated fetal bovine serum (FBS, Gibco, CA), 2 mM glutamine, 100 U/mL penicillin, and 100 μg/mL streptomycin in 5% CO_2_ at 37°C. The culture medium was changed every 48 h, and serum‐free medium was used when the cells reached 40% confluence. The cells were starved for 24 h and then were cultured with normal‐ (NG, 5.6 mM), high‐ (HG, 30 mM) glucose, and normal glucose plus mannitol (5.6 mM glucose +24.4 mM mannitol) medium for 72 h. The *vitro* experiments were repeated at least three independent times. The following compounds were used to treat HK‐2 cells: 100 μg/mL CHX (MCE, HY‐12320); 10 nmol/mL MG‐132 (MEC; HY‐13259).

### 2.3 Clinical specimens

Human renal tissues were obtained from patients during surgical nephrectomy. The samples were collected from kidney regions unaffected by the tumour (at least 5 cm from the tumour, i.e., the non‐tumour tissues at the distal margin) after the performance of conventional renal carcinoma nephrectomy at the First Affiliated Hospital of Zhengzhou University. The renal tissues were categorized into three groups based on the results of clinical evaluations and histopathology judgement. First, the negative control group (NC, *n* = 32) was established involving individuals with normal glucose tolerance (NGT) and without evident histopathological changes in their kidneys. Second, the diabetes mellitus (DM) group (DM, *n* = 22) consisting of patients with type 2 diabetes and albumin to creatinine ration (ACR) < 30 mg/g and without evident histopathological changes in their kidneys was established; lastly, the DKD group (DKD, *n* = 15) comprising type 2 diabetes patients with ACR > 30 mg/g, following subjection to fundus microscopy and kidney histopathology analyses conducted to confirm the presence of DKD, was established. Additionally, human plasma samples were collected from another set of healthy individuals with NGT (NC, *n* = 56), patients with DM (type 2 diabetes) but without DKD (DM, *n* = 44), and patients with DKD (DKD, *n* = 53); urinary sediment samples were collected from healthy individuals with NGT (NC, *n* = 20), patients with DM (type 2 diabetes) but without DKD (DM, *n* = 28), and patients with DKD (DKD, *n* = 36). These patients' characteristics are shown in Tables S1, S3, and S4.

### 2.4 Mouse model

C57BL/6J mice were obtained from Beijing Charles river (Beijing, China). Six‐week‐old male C57/BL6J mice were maintained on a normal chow diet and housed in the room at 22–25°C with a 12 h light/dark cycle schedule. After 2 weeks, the fasting mice for 12 h were intraperitoneally injected with streptozotocin (STZ; 50 mg/kg, in 0.05 M citrate buffer, pH 4.5; Sigma‐Aldrich) for five consecutive days to establish diabetic mouse model the model. The control mice were injected with citrate buffer. Fasting blood glucose level was measured at 72 h after STZ injection, diabetes was successfully established with blood glucose level > 16.7 mmol/L for three consecutive tests. The body weight and blood glucose level were recorded every 4 weeks for a period of 16 weeks.

### 2.5 Isolation of renal tubules

Mice were euthanized through CO_2_ inhalation and the fresh kidneys were immediately collected and washed in sterile Krebs–Henseleit saline (KHS; pH 7.4) with 95% O_2_/5% CO_2_. Afterwards, the renal cortex was collected, minced, and mixed in 30 ml KHS solution containing 1 mg/mL collagenase (Type I; Invitrogen, CA). The solution was aerated with 95% O_2_/5% CO_2_ during incubation at 37°C for 30 min and filtered through a 210 μm mesh sieve (Fisher Scientific, MA). The mixed renal cortexes solution was added to the test tube containing 30 ml of 45% Percoll (Sigma‐Aldrich) solution and 5 ml of 90% Percoll solution, and was then centrifuged for 30 min (20,000 × *g*, 4°C). The third layer of the solution was aspirated and centrifuged again at 1500 rpm for 2 min at 23–25°C to remove the Percoll solution. The renal tubules precipitate was then collected for subsequent experiments.

### 2.6 RNA microarray analysis

HK‐2 cells were cultured in a medium with 5.6 mM and 30 mM glucose concentration for 72 h. The RNA profiles of three NG and three HG groups were analysed using Arraystar Human lncRNA and mRNA arrays following the manufacturer's instructions. Quantile standardization was performed on the original data using the GeneSpring GX v12.1 software (Agilent Technologies, CA). LncRNAs and mRNAs with fold change ≥2.0 and *p‐*values <0.05 were considered differentially expressed. All microarray analyses were performed by KangChen Bio‐tech (Shanghai, China). RNA microarray data have been submitted to the GenBank databases (Gene Expression Omnibus) under accession number GSE158534. Addresses are as follows: GenBank http://www.ncbi.nlm.nih.gov.

### 2.7 Isolation of cytoplasmic and nuclear RNA

Cytoplasmic and nuclear RNA were isolated and purified using the Cytoplasmic & Nuclear RNA Purification Kit (Norgen Biotek, Belmont, CAN) according to the manufacturer's instructions. The first‐strand cDNA was generated using M‐MuLV Reverse Transcriptase (Sangon Biotech, Shanghai, China). Real‐time PCR was performed in the StepOne Real‐Time PCR System using SYBR Green (Sangon Biotech, Shanghai, China). The primers used for PCR are listed in Table S5. The relative expression of the target genes was calculated using the CT method (2^−△△CT^) and the data were normalized to *GAPDH*.

### 2.8 Cell transfection

The CDS of *TXNIP* and RNA binding motif protein X‐linked (*RBMX*) were cloned into pcDNA3.1 vector (GeneChem, Shanghai, China) respectively. Human *PWARSN* was subcloned into the lentiviral vector pLenti‐CMV‐MCS‐PURO (HANBIO, Shanghai, China).

Interference of *PWARSN* was conducted using the RiboTM Smart Silencer designed and synthesized in Ribobio (Guangzhou, China). Each RiboTM Smart Silencer contains three siRNA and three antisense oligonucleotides (ASOs) targeting different sequences. siRNA for human *TXNIP* was designed and synthesized by GenePharma (Shanghai, China). Three siRNA targeting different sequences for RBMX were designed and synthesized in Ribobio (Guangzhou, China). siRNA‐targeted indicated genes are listed in Table S5. These plasmids and vectors were transfected into HK‐2 cells using lipofectamine 2000 (Invitrogen, CA) according to the manufacturer's instructions.

For miR‐372‐3p studies, synthetic miR‐372‐3p mimics and inhibitors (Sangon Biotech, Shanghai, China) were transfected into HK‐2 cells following the manufacturer's instructions.

### 2.9 CRISPR/Cas9

Two guide RNAs (gRNA‐A1:5‐TCGTTGTGACACTACTGAGT‐AGG‐3, gRNA‐A2: 5‐CCGATTCAACTTAAAAAGCG‐AGG‐3 and gRNA‐B1:5‐ TTTCAGAGGCATCAACTAAG‐TGG‐3, gRNA‐B2:5‐ GATGTACAACCTAACCCTCA‐AGG‐3) targeting the genome sequence of *PWARSN* at exon 1 (E1) were designed in silico using the CRISPR Design Tool (http://crispor.tefor.net/crispor.py). Then gRNA‐A1‐U6 > gRNA‐A2‐U6 > gRNA‐B1‐U6 > gRNA‐B2 DNA fragment was obtained, which was ligated cloned into the vector pPB‐U6 > MCS‐CAG > hCas9 by T4 DNA ligase (Cyagen, Guangzhou, China). Enzyme digestion and sanger sequencing confirmed that the plasmid was right. The gRNA and lentivirus‐Cas9 were transcribed into mRNA in vitro. HK‐2 cells were plated at 1× 10^5^ cells per well and transfected with plasmids. Then HK‐2 cells were selected with hygromycin and puromycin and single‐cell colonies were cultured and verified for genome deletion by PCR and sanger sequencing. The PCR primers used are as follows: Primers for region 1: F: AGCAAAAACACCCTTTACCTGG; R: AGTGCAATACTACACCTGCCA; Primers for region 2: F: TCTTGCATGGTGAGATGGGG; R: TCAAGGCTGGCACAACGTAA; Primers for region 3: F: AGCAAAAACACCCTTTACCTGG; R: TCAAGGCTGGCACAACGTAA.

### 2.10 Adeno‐associated virus9 transduction of *PWARSN*

The full length of *PWARSN* was inserted into the pHB AAV‐CMV‐MCS‐PURO vector (HANBIO, Shanghai, China). Adeno‐associated virus9 (AAV9) (1 × 10^12^ vg/ml) harbouring either *PWARSN* or control sequence and saline was injected into the 5–6 sites of the left renal cortex evenly using a 100‐μl Hamilton syringe (120 μl/mouse) in eight‐week wild‐type mice according to our previous studies.^23^, ^24^ Double in situ hybridization (ISH) (*PWARSN*) and immunofluorescence staining (AQP1, 20,333‐1‐AP, Proteintech, Wuhan, China) were used to determine the distribution of AAV9‐*PWARSN* in the renal cortex. The qRT‐qPCR assay was used to detect *PWARSN* expression after transfection.

### 2.11 Quantitative real‐time PCR

Total RNA was isolated using the trizol reagent (Invitrogen, CA). The first‐strand cDNA was generated using M‐MuLV Reverse Transcriptase (Sangon Biotech, Shanghai, China). Real‐time PCR was performed in the StepOne Real‐Time PCR System using SYBR Green (Sangon Biotech, Shanghai, China). miRNAs were extracted using an miRNA isolation kit (Invitrogen, CA). The primers used for PCR are listed in Table S5. The relative expression of the target genes was calculated using the CT method (2^−△△CT^) and the data were normalized to those of *β‐actin* and *U6*.

### 2.12 Fluorescence in situ hybridization

The probes and kits used for fluorescence in situ hybridization (FISH) were designed by and purchased from GenePharma (Shanghai, China). For double FISH, Cy3‐ and FITC‐labelled cDNA probes (Table S5), RBMX (14794, Cell Signaling Technology) antibodies or TXNIP (14715, Cell Signaling Technology) antibodies were added. The samples were counterstained with DAPI and observed using confocal or fluorescence microscopy (Olympus, Japan).

### 2.13 LncRNA–TXNIP co‐expression network

The lncRNA–TXNIP co‐expression network was built using the LncTar database (http://www.cuilab.cn/lnctar),^25^ Starbase (http://starbase.sysu.edu.cn/)^26^, ^27^ and RNA microarray results. Confirmed the median score as the expression value of *TXNIP* based on the standardized data and different transcripts of *TXNIP*. Calculated the correlation coefficient between the normalized data of the selected lncRNAs and *TXNIP*. An absolute Pearson correlation coefficient (PCC) value of ≥0.90 and a *p*‐value of <0.05 were recommended and retained for further analysis. Established the network using Cytoscape software.^28^ Pink node is lncRNA; the Green node is TXNIP. Solid lines represent a positive correlation and dotted lines represent a negative correlation. The list of the lncRNAs was showed in Table S6.

### 2.14 *PWARSN*–miRNAP–*TXNIP* co‐expression network

According to the ceRNA hypothesis, *PWARSN* competes for the same miRNA response elements, thereby regulating the de‐repression of all target genes of the respective miRNA family.^29^ The *PWARSN*‐miRNA network was predicted using a home‐made miRNA target prediction software from Arraystar based on TargetScan^30^ (http://www. targetscan.org) and miRDB^31^ (http://www.mirdb.org/) software. TXNIP related miRNAs were downloaded using the miRTarbase^32^ (http://mirtarbase.mbc.nctu.edu.tw/php/index.php) software. Then selected the same miRNAs between *PWARSN*‐miRNA network and TXNIP‐miRNA network. Finally, the regulatory networks of *PWARSN*‐miRNA‐TXNIP were constructed using the Cytoscape 3.7.1 software.^28^

### 2.15 Luciferase reporter assays

The 3′‐UTRs of the *PWARSN* and *TXNIP* cDNA fragments containing miR‐372‐3p binding sites were amplified using PCR and subcloned downstream of the luciferase gene in the pmiRGLO luciferase vector (GenePharma, Shanghai, China). pmirGLO‐*PWARSN*‐WT or pmirGLO‐*PWARSN*‐MUT, and pmirGLO‐TXNIP‐WT or pmirGLO‐TXNIP‐MUT were co‐transfected with the miR‐372‐3p mimics or inhibitor into HK‐2 cells.

To investigate the effects of RBMX on TXNIP promoter activity, *PWARSN* or *RBMX*‐overexpressing plasmid were co‐transfected with the control pGL3 plasmid or the pGL3‐TXNIP truncated promoter regions firefly luciferase constructs (GenePharma, Shanghai, China). After 48 h, the relative luciferase activity was normalized to renilla luciferase activity using a Dual‐Luciferase Reporter Assay (Promega, WI) according to the manufacturer's instructions.

### 2.16 RNA immunoprecipitation

HK‐2 cells were transfected with miR‐372‐3p mimics and NC mimics for 48 h. RNA immunoprecipitation (RIP) was performed using an AGO2 antibody (ab186733, Abcam) according to the manufacturer's instructions. The RNA fraction isolated was quantified and assessed using NanoDrop 2000 (Thermo Scientific). RNA was then reverse‐transcribed into the first‐strand cDNA using reverse transcriptase and random primers, followed by second‐strand cDNA synthesis using DNA polymerase I and RNase H. RNA enrichment was detected using qRT‐PCR. The relative expression of the target genes (miR‐372‐3p and *PWARSN*) was calculated using the CT method (2^−△△CT^).

### 2.17 RNA interactome

HK‐2 cells were collected (8 × 10^7^ cells) and added to 400 μl RIP lysis buffer. Total RNA was isolated using the trizol reagent. The *PWARSN* plasmid was constructed and linearized using NdeI and BamHI according to the sequence map and base information of the mic‐RNA sequence. The gel of interest was recovered and purified. The target RNA was labelled with biotin and bound to the magnetic beads labelled with streptavidin to form an RNA–RNA binding interaction system. Total RNA was extracted, purified, and reversed to obtain the template cDNA. The expression of miR‐372‐3p was detected using qRT‐PCR.

### 2.18 Chromatin Isolation by RNA Purification (ChIRP) analysis

The antisense oligo probes of *PWARSN* were designed, synthesized, and modified with biotin‐TEG at the 3′ ends by KangChen Bio‐tech (Shanghai, China). HK‐2 cells (1 × 10^8^ cells per sample) were then crosslinked with 3% formaldehyde solution at 25°C for 10 min. Lysis buffer was added to the solution and was centrifuged to remove the supernatant. Next, two volumes of the hybridization buffer were added and mixed. This was combined with the biotin‐labelled probe and the magnetic beads for 30 min. After overnight hybridization at 37°C, elution buffer (100 μl) was added to resuspend the magnetic beads, and nuclease benzonase was added to react at 37°C for 1 h. After further reaction at 95°C for 30 min, the suspension was crosslinked with 0.1% SDC and 10% TCA and precipitated at 4°C for 2 h. ABC (200 ml; 100 mM, pH 8.5, 0.1% SDC) was added to dissolve the precipitate, and 5 mm TCEP, 10 mM IAA, 0.5 μg Trypsin, and TFA were subsequently added to terminate the digestion. Precipitation was desalinated for MS detection. The enzymatic hydrolysates were separated using nano‐UPLC and analysed online using a Q‐Exactive mass spectrometer (Thermo Finnigan). The MS raw data were quantitatively analysed using MaxQuant.

### 2.19 RIP‐seq and qRT‐PCR

After the isolation of HK‐2 cellular nuclei, the extract was lysed in polysome lysis buffer for 30 min in an ice bath and centrifuged. IgG and RBMX antibodies were diluted to 1:1000 in a buffer. The RNA levels were then quantified using Qubit 2.0. The stranded RNA sequencing library was constructed by using KC‐DigitalTM Stranded mRNA Library Prep Kit for Illumina (DR08502, Seqhealth Co, Wuhan, China) following the manufacturer's instructions. The kit eliminates duplication bias in PCR and sequencing steps, by using a unique molecular identifier (UMI) of 8 random bases to label the pre‐amplified cDNA molecules. The library products corresponding to 200–500 bps were enriched, quantified and finally sequenced on Novaseq 6000 sequencer (Illumina) with a PE150 model. Then the original data were filtered by Trimmomatic (version 0.36), low‐quality reads were discarded and the reads contaminated with adaptor sequences were trimmed. Clean Reads were further treated with in‐house scripts to eliminate duplication bias introduced in library preparation and sequencing. Gene expression was then quantified, and the co‐precipitated RNAs were detected using qRT‐PCR. The gene‐specific primers used for *PWARSN* and *TXNIP* detection are listed in Table S5.

### 2.20 RNA pull‐down assay

Cell lysates were incubated with streptavidin‐coated magnetic beads (Invitrogen) following the manufacturer's instructions. The enriched RBMX in the captured fractions were eluted from the packed beads and analysed by western blotting.

### 2.21 Chromatin immunoprecipitation

HK‐2 cells cultured in high glucose medium were crosslinked with formaldehyde for 10 min, and the reaction was terminated using 0.125 M glycines. The solution was sonicated to generate 300–600 bp DNA fragments and purified using protein‐A‐coated magnetic beads for 1 h. The samples were incubated with RBMX and H3K9me3 antibodies overnight. The binding of RBMX and H3K9me3 to TXNIP promoter or IgG was quantified using qRT‐PCR with the specific primers listed in Table S5.

### 2.22 Co‐immunoprecipitation

HK‐2 cells were collected and lysed in an immunoprecipitation buffer containing a complete protease inhibitor cocktail (Roche, Switzerland). Then cell lysates were centrifuged at 10,000 × *g* for 30 min at 4°C, and the supernatant was collected and incubated with 1.0 μg IgG and 20 μl A/G‐beads for 1 h at 4°C. Then lysates were centrifuged at 2000 × *g* and 4°C for 5 min, and the supernatant was collected and incubated with 1–10 μl (0.2–2 μg) of antibody or ubiquitin antibody at 4°C overnight. Further incubation with 80 μl A/G‐beads at 4°C for 2 h was performed. The immunoprecipitated complexes were collected after centrifugation at 1000 × *g* and 4°C for 5 min. The immunocomplexes were then washed with immunoprecipitation lysis buffer three times, boiled in SDS sample buffer, and subjected to SDS‐PAGE and western blotting.

### 2.23 Western blotting

HK‐2 cells and renal tubule tissue were lysed in RIPA buffer (Solarbio, Beijing, China) with protease inhibitor using the following primary antibodies: anti‐TXNIP (14715, Cell Signaling Technology), anti‐NLRP3 (15101, Cell Signaling Technology), anti‐caspase‐1 (24232/3866, Cell Signaling Technology), anti‐cleaved IL‐1β (83186/63124, Cell Signaling Technology), anti‐ASC (sc‐514414, Santa Cruz), anti‐GSDMD (NBP2‐33422, Novus Biologicals, CO/ab219800, Abcam), anti‐cleaved caspase‐1 (4199/89332, Cell Signaling Technology), anti‐RBMX (14794, Cell Signaling Technology), anti‐β‐actin (sc‐81,178, Santa Cruz), and anti‐Histone3 (17168‐1‐AP, Proteintech, Wuhan, China). Western blotting was performed after incubation with a horseradish peroxidase‐conjugated anti‐rabbit/anti‐mouse secondary antibody (D110011/D110087, Sangon Biotech, Shanghai, China).

### 2.24 Metabolic data in mice

Twenty four hours UTP was measured according to the previous study.^16^ Blood samples were collected from the orbital vein to measure Scr (Rayto Life and Analytical Sciences Co, Shenzhen, China), BUN (Roche, Switzerland) and KIM‐1 (USCN KIT INC, Wuhan, China) level (Thermo Scientific, MA) according to the manufacturer's instructions.

### 2.25 Measurement of intracellular ROS and mitochondrial ROS accumulation

The adherent cells were incubated in the culture medium with 10 mmol/L 2′7‐dichlorodihydrofluorescein‐diacetate (H2DCFDA) probe (K936, Biovision, VA) for 30 min at 37°C protected from light, and the medium was removed and then visualized using fluorescence microscopy. The levels of mitochondrial ROS (mitoROS) were measured using MitoSOX Red (M36008, Invitrogen) according to the manufacturer's protocol and as previously described. The HK‐2 cells and mRTECs were incubated with MitoSox (5 μM) at 37°C for 20 min protected from light and then visualized using fluorescence microscopy (Olympus, Japan).

Tissue mitochondrial extraction kits (C3606, Beyotime, Shanghai, China) were used for mitochondrial isolation of renal tubules according to the manufacturer's instructions and as previously described. Then the levels of mitochondrial ROS were measured using MitoSOX Red (M36008, Invitrogen) and analysed using a fluorescence microplate reader.

### 2.26 Propidium iodide staining

Propidium iodide (PI, Solarbio) staining was performed. After specific treatment, 1/10 volume of the PI solution (33.4 μg/ml) was added to the medium, followed by incubation at 37°C for 10–20 min. The cells were washed twice with a suitable buffer and observed using fluorescence microscopy (Olympus, Japan).

### 2.27 Immunofluorescence and immunohistochemistry staining

Immunofluorescence and immunohistochemistry staining were performed on paraffin‐embedded or OCT‐embedded tissues fixed by 4% paraformaldehyde using standard techniques. The primary antibodies were used as followed: anti‐TXNIP antibody (1:200, 14715, Cell Signaling Technology), anti‐NLRP3 (15101, Cell Signaling Technology), anti‐caspase‐1 (24232/3866, Cell Signaling Technology), anti‐ASC (sc‐514414, Santa Cruz), anti‐GSDMD (NBP2‐33422, Novus Biologicals/ab219800, Abcam). The cell nuclei were incubated with 4′,6‐diamidino‐2‐phenylindole (DAPI) (Sigma‐Aldrich). The images were examined using fluorescence microscopy or light microscopy (Olympus, Japan).

### 2.28 Microscopy

Sections for kidney pathology were stained using haematoxylin and eosin (HE), periodic acid‐schiff (PAS), and Masson trichrome (Masson) and were examined using light microscopy (Olympus, Japan). The ultrastructure of renal tubule and HK‐2 cells were observed using transmission electron microscopy (HT7800; Hitachi, Japan) and scanning electron microscopy (SU8100; Hitachi, Japan).

### 2.29 Live‐cell imaging and analysis

HK‐2 cells were seeded in a 96‐well plate and incubated for 72 h. The images of the HK‐2 cell were automatically captured every hour using a phase‐contrast microscope. The images were obtained using IncuCyte image analysis software (IncuCyte S3 Software, Essen BioScience).

### 2.30 Statistics

Statistical analysis was performed using the SPSS v23.0 software and GraphPad Prism 6. Continuous variables are expressed as means ± SD median (interquartile range) of at least three biological replicates (unless specified otherwise in Figure legends). The normality of data was tested using the Shapiro–Wilk test. For normally distributed continuous variables, unpaired two‐tailed Student's *t*‐test was used for two independent groups, and one‐way analysis of variance (ANOVA) followed by LSD‐t or Bonferroni's test was used for multiple groups. Mann–Whitney *U* test and Kruskal‐Wallis test were used for non‐normal distribution data. Chi‐square test was used for comparison between sexes in patients. Spearman correlation analysis was used to analyse the associations between *PWARSN*, TXNIP mRNA and NLRP3 mRNA levels. ROC curve analysis was performed to analyse the power of indicated variables in the diagnosis of patients with DKD. *p‐*values <0.05 were considered significant.

## 3 RESULTS

### 3.1 TXNIP induces NLRP3 inflammasome activation and accelerates tubular cell pyroptosis in DKD

**FIGURE 1:**
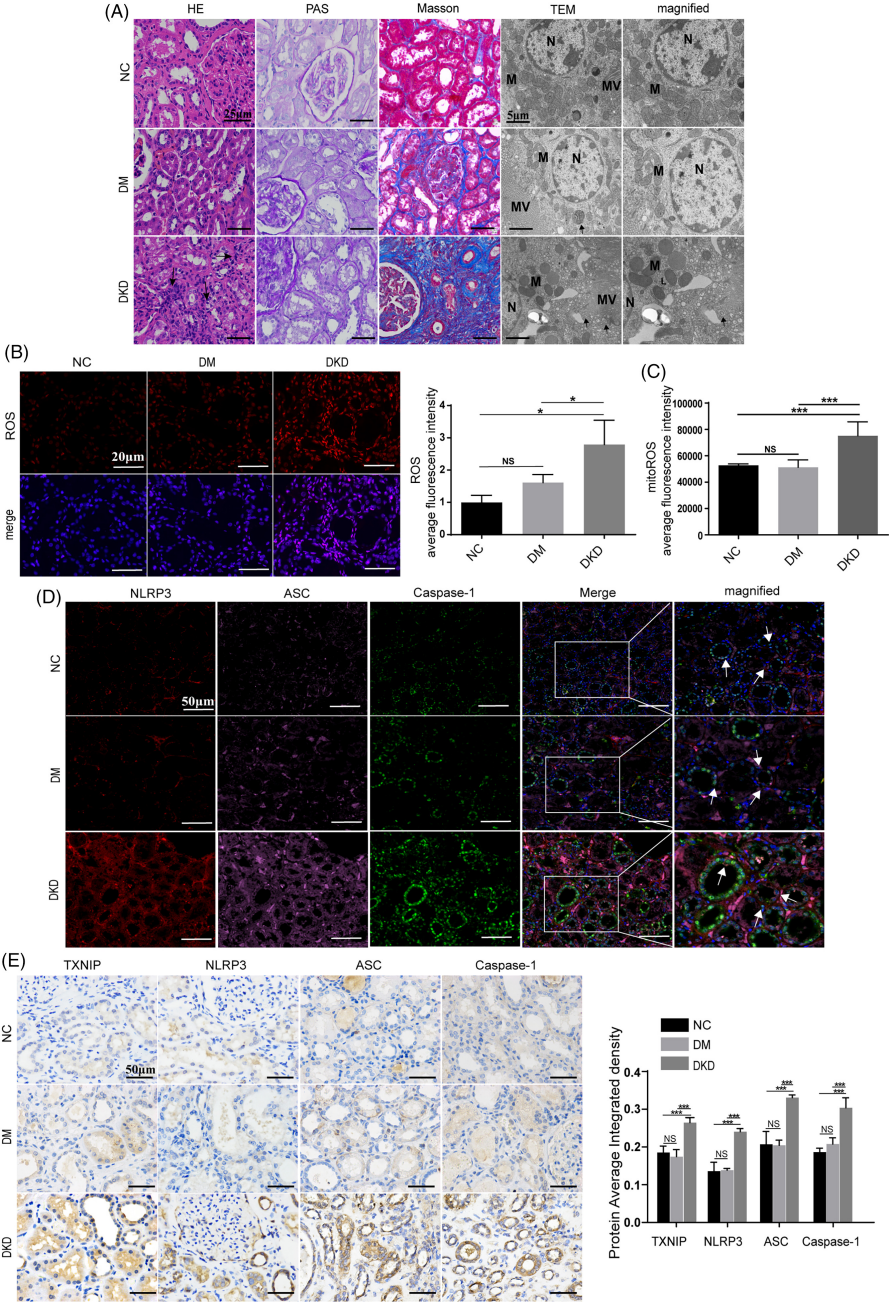
TXNIP induces NLRP3 inflammasome activation and accelerates tubular cell pyroptosis in DKD. (A‐E) Kidney samples were obtained from patients with NGT (NC group), patients with DM but without DKD (DM group), and patients with DKD (DKD group). NGT: normal glucose tolerance; DM: diabetes mellitus; DKD: diabetic kidney disease. (A) Representative images viewed using HE (black arrows: inflammatory cells), PAS, Masson's staining and TEM in NC, DM and DKD groups (DKD: pyroptosis cells with disrupted microvilli, few mitochondria with hardly recognizable structures, autophagic vacuoles, and lipid droplets. N: nucleus; M: mitochondria; MV: microvilli; black arrows: autophagic vacuoles; L: lipid droplets). HE: haematoxylin and eosin; PAS: periodic‐acid schiff; TEM: transmission electron microscopy. Scale bar, 25 μm, 5 μm. (B and C) Intracellular ROS (B) and mitochondrial ROS (C) levels were assessed using immunofluorescence microscopy. Scale bar, 20 μm. (D) Co‐localization of NLRP3 (red), ASC (pink), and Caspase‐1 (green) in the renal tissues viewed using scanning immunofluorescence microscopy in the NC, DM and DKD groups. Scale bar, 50 μm. (E) The expression levels of TXNIP, NLRP3, ASC, and Caspase‐1 in the renal tissues viewed using immunohistochemical staining in the NC, DM and DKD group. *n* = 3 per group. Scale bar, 50 μm. The data were obtained from at least three independent experiments. Data are presented as the means ± SD, and significance was determined using one‐way ANOVA (B, C, E) for multiple groups. (*p < 0.05; **p < 0.01; ***p < 0.001; NS: not significant).

We collected renal tissue from patients who had undergone nephrectomy to detect the expression levels of TXNIP and NLRP3 inflammasome. The samples included those patients with normal glucose tolerance (NGT), patients with type 2 diabetes mellitus (DM) but without DKD, and patients with DKD. Significant inflammatory cell infiltration, mitochondria swelling, tubular basement membrane thickening and tubulointerstitial fibrosis were observed in the kidneys of patients with DKD (Figure 1A). Immunofluorescence analysis of renal tissue from patients showed remarkable elevation levels of mitochondrial reactive oxygen species (mitoROS) and ROS, accompanied with increases co‐localization of NLRP3 inflammasome (NLRP3, ASC and Caspase‐1) in patients with DKD (Figure 1B–D). Furthermore, immunohistochemistry analysis also confirmed that the levels of TXNIP, NLRP3, ASC and Caspase‐1 were significantly increased (Figure 1E). Additionally, similar pathological findings were observed in the kidneys of diabetic mice (Figure S1).

Next, we assessed the role of TXNIP in activating the NLRP3 inflammasome and pyroptosis in PTECs (HK‐2 cells) cultured under normal glucose (NG) and high glucose (HG) conditions. Dynamic death and ultrastructural changes were identified through IncuCyte assays, which showed that the cytoplasmic distribution was translocated to and accumulated on the plasma membrane of HK‐2 cells in the HG group, subsequently leading to cell swelling and bubbling and even rupturing, as pyroptosis progressed (Figure S2A). Increased pores in the plasma membrane and cell rupture of HK‐2 cells were observed by transmission electron microscopy (TEM) and scanning electron microscopy (Figure S2B). Furthermore, *TXNIP* deficiency reduced pyroptosis‐related protein levels in the HG group, whereas its overexpression increased pyroptosis‐related protein levels in the NG group (Figure S2C–E). Collectively, these findings demonstrate that TXNIP‐induced NLRP3 inflammasome activation and subsequent cell pyroptosis are involved in the progression of DKD.

### 3.2 The PTEC‐specific lncRNA *PWARSN* is pathologically associated with DKD

**FIGURE 2:**
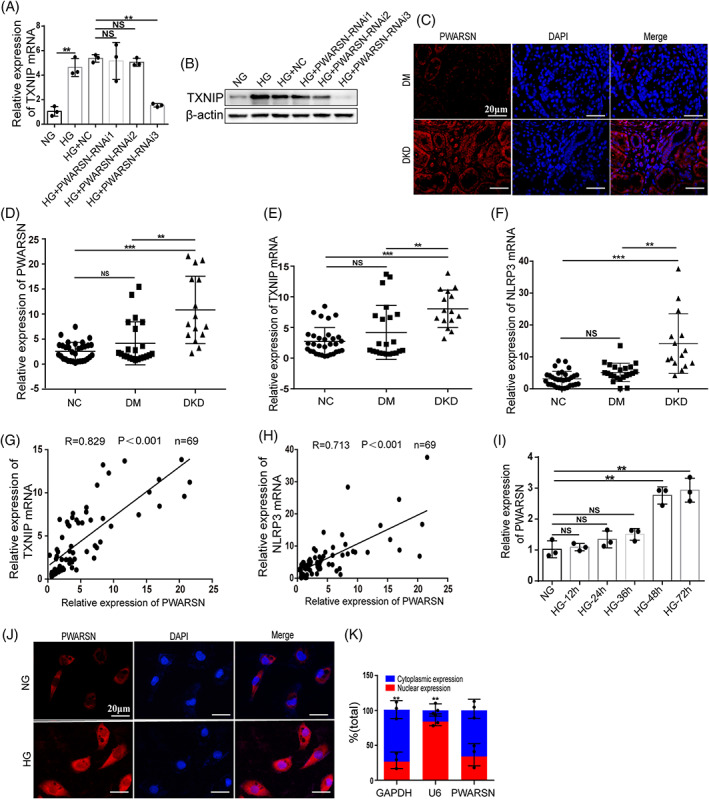
The PTEC‐specific lncRNA *PWARSN* is pathologically associated with DKD. (A and B) TXNIP mRNA (A) and protein (B) levels in *PWARSN*‐knockdown HK‐2 cells by qRT‐PCR and western blotting. (C) FISH images showing upregulated *PWARSN* were primarily in the renal tubules of patients with DKD (*n* = 3 per group). FISH: fluorescense in situ hybridization. Scale bar, 20 μm. (D–F) The expression of *PWARSN* (D), TXNIP mRNA (E) and NLRP3 mRNA (F) in isolated renal tubules from patients with NGT (NC, *n* = 32), patients with DM but without DKD (DM, *n* = 22), and patients with DKD (DKD, *n* = 15) by qRT‐PCR. NGT: normal glucose tolerance; DM: diabetes mellitus; DKD: diabetic kidney disease. (G and H) Relative TXNIP mRNA (G) and NLRP3 mRNA (H) levels in the renal tubules of clinical patients were positively correlated with that of *PWARSN* (G, *R* = 0.829, ***p < 0.001; H, *R* = 0.713, ***p < 0.001; *n* = 69). (I) Relative expression of *PWARSN* in NG‐ and HG‐treated HK‐2 cells cultured at different periods by qRT‐PCR. (J) FISH for *PWARSN* in NG‐ and HG‐treated HK‐2 cells for 72 h. FISH: fluorescense in situ hybridization. Scale bar, 20 μm. (K) qRT‐PCR for the abundance of *PWARSN* in either the cytoplasm or nucleus in HG‐treated HK‐2 cells for 72 h. The data were obtained from at least three independent experiments. Data are presented as the means ± SD, and significance was determined using one‐way ANOVA (A, I), Kruskal‐Wallis Test (D, E, F) for multiple groups, and Spearman correlation analysis (G, H) (*p < 0.05; **p < 0.01; ***p < 0.001; NS, not significant).

To explore the molecular mechanisms of TXNIP/NLRP3 signaling activation in renal tubules of DKD, we performed whole transcriptome RNA‐microarray analysis in HK‐2 cells treated with NG and HG for 72 h (Figure S3A–C). TXNIP mRNA was prominently upregulated in cells treated with HG according to microarray results. This was confirmed by quantitative real‐time PCR (qRT‐PCR) (Figure S3D). Eleven TXNIP‐associated lncRNAs were identified in the constructed lncRNA–TXNIP co‐expression network (Figure S3E), and five dysregulated lncRNAs were identified after validation by qRT‐PCR. Intriguingly, lncRNA *PWARSN* (also known as *NR_0221011*) was markedly increased in the HG group (Figure S3F). As a 1797‐nucleotide (nt) lncRNA encoded by human chromosome 15 (http://genome.ucsc.edu/), *PWARSN* is poorly conserved across species and exhibits little coding capability (Figure S3G–I). Notably, *PWARSN* knockdown, achieved by the mixture of short interfering RNAs and antisense oligonucleotides in HK‐2 cells exerted a significant effect on the mRNA and protein levels of TXNIP (Figure 2A, B, and Figure S3J). Thus, we chose *PWARSN* for further study.

To investigate the potential function and clinical significance of *PWARSN* in DKD, we first performed fluorescence in situ hybridization (FISH) assays in kidneys and found that *PWARSN* was primarily located in the renal tubules of patients with DKD (Figure 2C). Then, we evaluated the expression of *PWARSN*, TXNIP and NLRP3 in the isolated renal tubules from 32 patients with NGT, 22 patients with DM but without DKD and 15 patients with DKD (Table S1). qRT‐PCR showed that the levels of *PWARSN*, TXNIP mRNA, and NLRP3 mRNA were highly increased in patients with DKD compared to those of patients with NGT and DM. However, there was no significant difference in expression between patients with NGT and DM (Figure 2D–F). Notably, we identified strong correlations between *PWARSN* and TXNIP mRNA and between *PWARSN* and NLRP3 mRNA (Figure 2G and H), suggesting a critical association between *PWARSN* and TXNIP/NLRP3 in the renal tubules of DKD.

To further clarify the distribution and expression of *PWARSN* in different renal cell components, human PTECs, podocytes and mesangial cells were evaluated. After 72 h, HG induced the highest *PWARSN* expression in PTECs (Figure 2I) but had no effect on *PWARSN* expression in podocytes or mesangial cells (Figure S3K). Moreover, FISH and subcellular fractionation assays showed that *PWARSN* was distributed in both the cytoplasm and nucleus of PTECs (Figure 2J and K).

### 3.3 *PWARSN* triggers TXNIP/NLRP3‐induced pyroptosis of tubular epithelial cells both *in vitro* and *in vivo*

**FIGURE 3:**
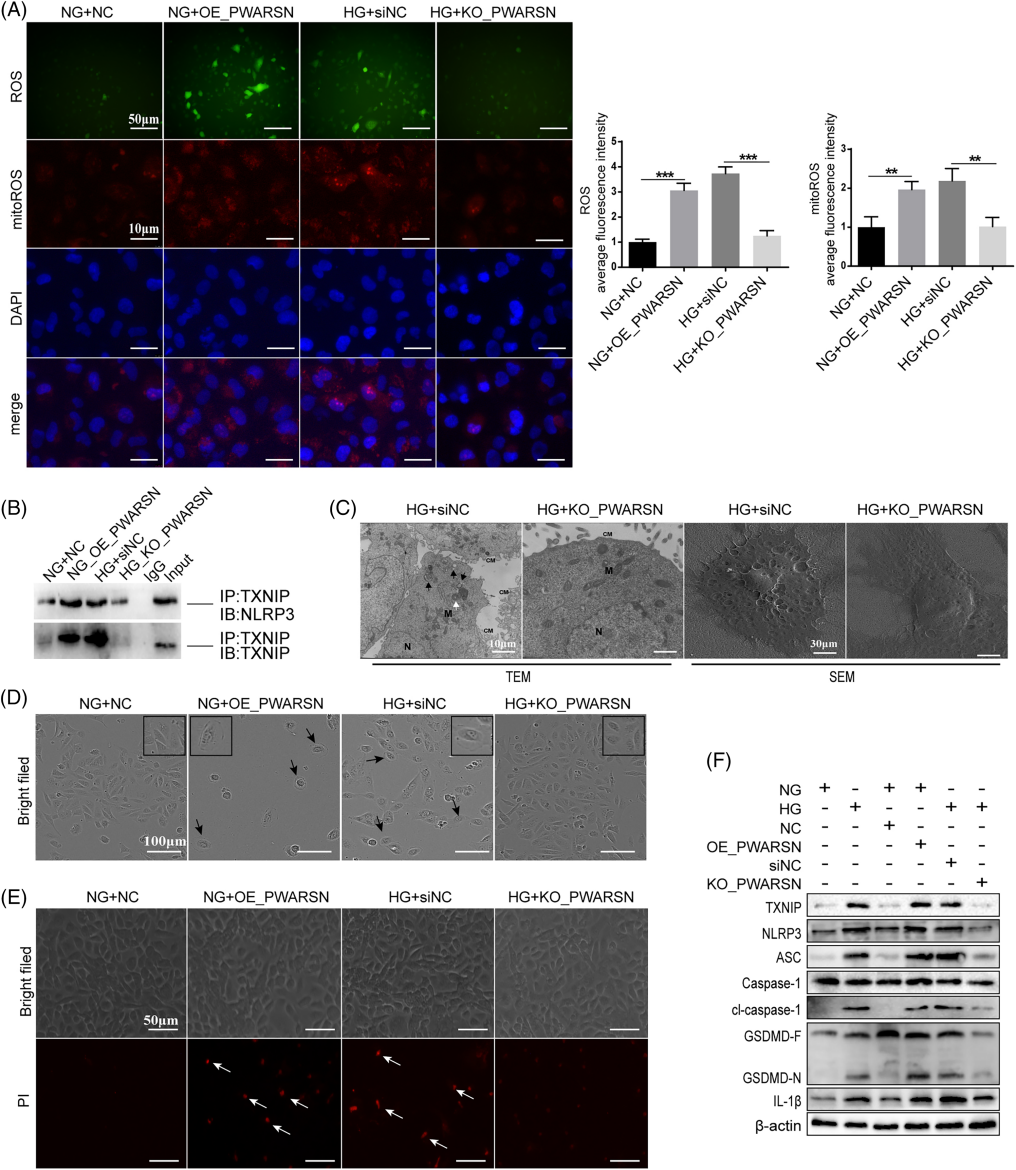
*PWARSN* triggers TXNIP/NLRP3‐induced pyroptosis of tubular epithelial cells under high glucose condition. (A) Overexpression of *PWARSN* promoted the accumulation of intracellular reactive oxygen species (ROS) and mitochondrial ROS in NG‐treated cells, whereas knocking out *PWARSN* prevented ROS generation in HG‐treated cells. Scale bar, 50 μm, 10 μm. (B) *PWARSN* overexpression enhanced the interaction between TXNIP and NLRP3 in NG‐treated HK‐2 cells, whereas its deficiency inhibited the interaction in HG‐treated cells using co‐immunoprecipitation assay. (C) Representative images of cell pyroptosis in *PWARSN*‐knockout cells by TEM and SEM. (TEM: HG‐treated cells showed decreased mitochondria with hardly recognizable structures (M), increased autophagosome (black arrows), multilamellar bodies (white arrows), and dissolved cell membrane (CM); SEM: Pores formation in plasma membrane were increased in HG‐treated cells). Scale bar, 10 μm, 30 μm. (D) Representative images of pyroptosis in dynamic live HK‐2 cells viewed using IncuCyte assays. The cells showed typical characteristics of pyroptosis such as swelling, bubbling and rupturing. Black arrows: pyroptotic cells. Scale bar, 100 μm. (E) Representative images of pyroptosis (red) in HK‐2 cells assessed using propidium iodide staining. White arrows: pyroptotic cells. Scale bar, 50 μm. (F) The levels of pyroptosis‐related proteins in *PWARSN*‐overexpressing and *PWARSN‐*knockout HK‐2 cells by western blotting. The data were obtained from at least three independent experiments. Data are presented as the means ± SD, and significance was determined using unpaired Student's *t*‐test (A) for two groups (**p < 0.01; ***p < 0.001).

To further explore the biological function of *PWARSN* in PTECs, we overexpressed *PWARSN* using a lentivirus system and knocked out *PWARSN* using the CRISPR‐Cas9 system in HK‐2 cells (Figure S4A–C). We first examined the effect of *PWARSN* on oxidative stress and TXNIP/NLRP3 signaling activation. The results showed that knocking out *PWARSN* downregulated the levels of ROS and mitoROS, TXNIP and pyroptosis proteins compared with the HG group; however, overexpression of *PWARSN* in KO cells abolished these effects (Figure S4D–F). Furthermore, knocking out *PWARSN* in HG decreased ROS and mitoROS generation, whereas overexpressing *PWARSN* in NG led to their accumulation (Figure 3A). Co‐immunoprecipitation assay showed that the TXNIP‐NLRP3 interaction was enhanced in *PWARSN*‐overexpressing cells but suppressed in *PWARSN*‐knockout cells (Figure 3B). Subsequently, overexpressing *PWARSN* promoted PTECs pyroptosis while *PWARSN* deficiency ameliorated cell pyroptosis. This was determined by scanning electron microscopy and TEM (Figure 3C), IncuCyte assays (Figure 3D), and propidium iodide staining (Figure 3E) in HK‐2 cells and was verified using western blotting (Figure 3F). Notably, knocking out *PWARSN* downregulated the levels of ROS and pyroptosis proteins; however, overexpression of *TXNIP* partially abolished these effects (Figure S4G, H).

**FIGURE 4:**
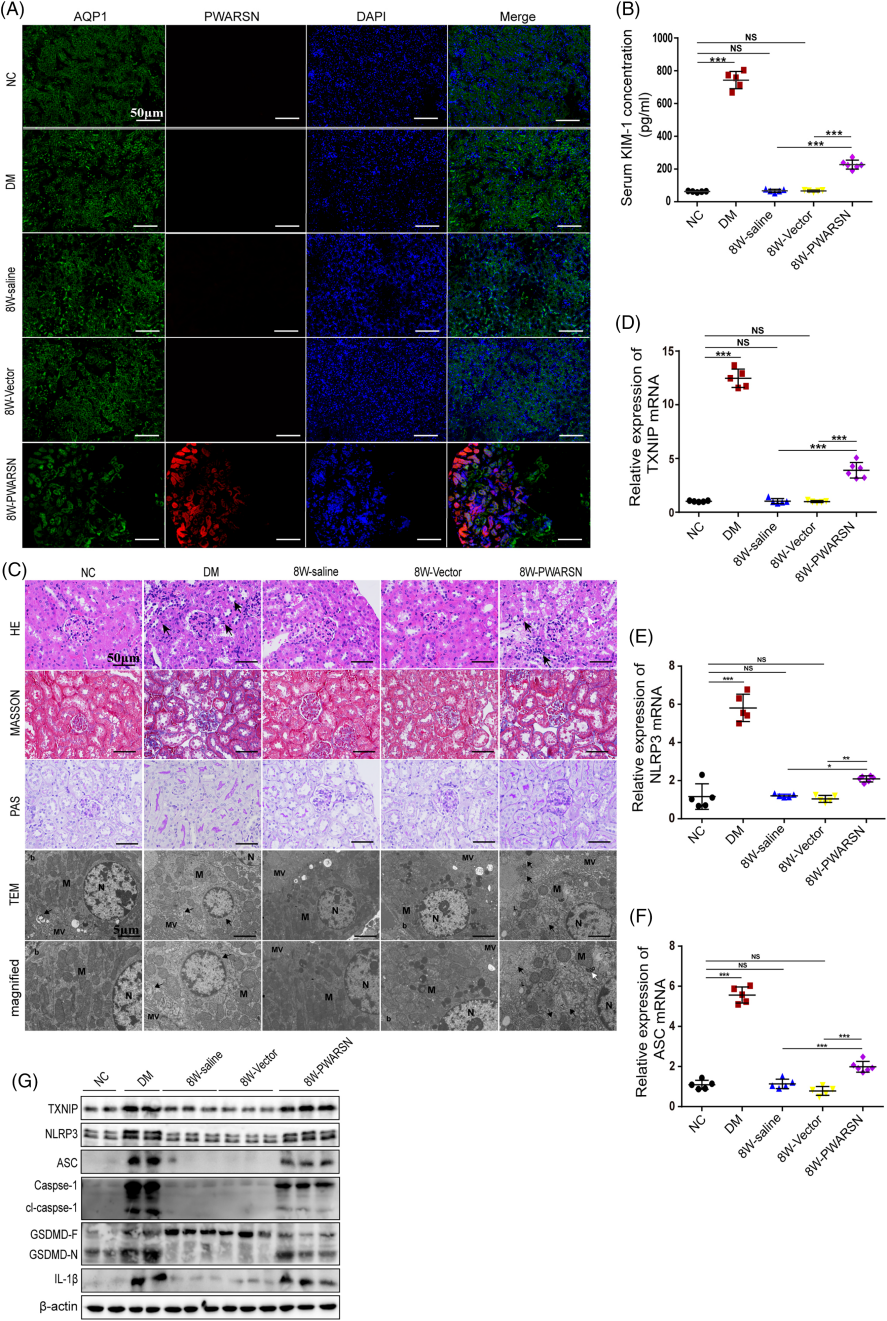
Delivery of human *PWARSN* contributes to tubular inflammation and mRTECs pyroptosis in wild‐type mice. (A) Representative immunostaining images for co‐localization of *PWARSN* (red) and renal tubule‐specific marker aquaporin 1 (AQP1, green) in the renal tubules of mice at 8 weeks after AAV9‐*PWARSN* injection. NC: normal control mice; DM: diabetic mice. Scale bar, 50 μm. (B) Serum kidney injury molecule 1 (KIM‐1) level in mice injected with AAV9‐*PWARSN* measured using ELISA assay. NC, *n* = 5; DM, *n* = 5; 8 W‐saline, *n* = 5; 8 W‐Vector, *n* = 5; 8 W‐*PWARSN*, *n* = 6. (C) Representative images of HE, Masson, PAS staining, and TEM in the renal samples of mice (HE: black arrows: inflammatory cells; TEM: mRTECs in 8W‐*PWARSN* mice showed swollen nucleus with disintegrating chromatin, vacuolized cytoplasm, decreased mitochondria with hardly recognizable structures (M), increased autophagosome (black arrows), multilamellar bodies (white arrows), lipid bodies (L), and disrupted and irregular microvillus (MV)). NC: normal control mice; DM: diabetic mice. Scale bar, 50 μm, 5 μm. (D–F) Relative Txnip mRNA, Nlrp3 mRNA and Asc mRNA levels in the renal tubules of mice injected with AAV9‐*PWARSN* by qRT‐PCR. NC, *n* = 5; DM, *n* = 5; 8 W‐saline, *n* = 5; 8 W‐Vector, *n* = 5; 8 W‐*PWARSN*, *n* = 6. NC: normal control mice; DM: diabetic mice. (G) The levels of Txnip and pyroptosis‐related proteins in the renal tubules of mice injected with AAV9‐*PWARSN* by western blotting. The data were obtained from at least three independent experiments. Data are presented as the means ± SD, and significance was determined using one‐way ANOVA (B, D, E, F) for multiple groups (*p < 0.05; **p < 0.01; ***p < 0.001; NS, not significant).

Considering the homologous sequence of human *PWARSN* has not been identified in mice or rats, we attempted to explore the influence of *PWARSN* by transfecting human *PWARSN* plasmid into mouse renal tubular epithelial cells (mRTECs). Excitingly, *PWARSN* enhanced TXNIP/NLRP3‐related pyroptosis in mRTECs (Figure S5). We then injected adeno‐associated virus9 carrying human *PWARSN* or control vector into the renal cortex of wild‐type C57/BL6J mice (Figure S6A) according to previous studies.^23^, ^24^ *PWARSN* was mostly distributed in the renal tubules at 8 weeks after the AAV9‐*PWARSN* injection (Figure 4A, and Figure S6B) and was accompanied by increases in the biochemical indexes of renal injury but showed no effect on body weight or blood glucose levels (Table S2; Figure 4B). Histological analysis by haemoxylin and eosin, periodic acid–Schiff, Masson's staining and TEM assays showed an increase in proximal tubular cell atrophy, tubular basement membrane thickening and a markedly changed ultrastructure of pyroptotic mRTECs (mitochondria swollen and crista lost) in the AAV9‐*PWARSN*‐injected mice (Figure 4C), as well as the promotion of mitoROS and ROS accumulation visualized by immunofluorescence analysis (Figure S6C, D). Additionally, *PWARSN* elicited an additive upregulation of the expression of Txnip and pyroptosis‐related proteins, at both mRNA and protein levels (Figure 4D–G). Overall, these results suggest that *PWARSN*, a non‐conserved human lncRNA, performs a conserved function in regulating TXNIP/NLRP3‐induced cell pyroptosis of renal tubules in DKD.

### 3.4 *PWARSN* sponges miR‐372‐3p in the cytoplasm to regulate TXNIP

**FIGURE 5:**
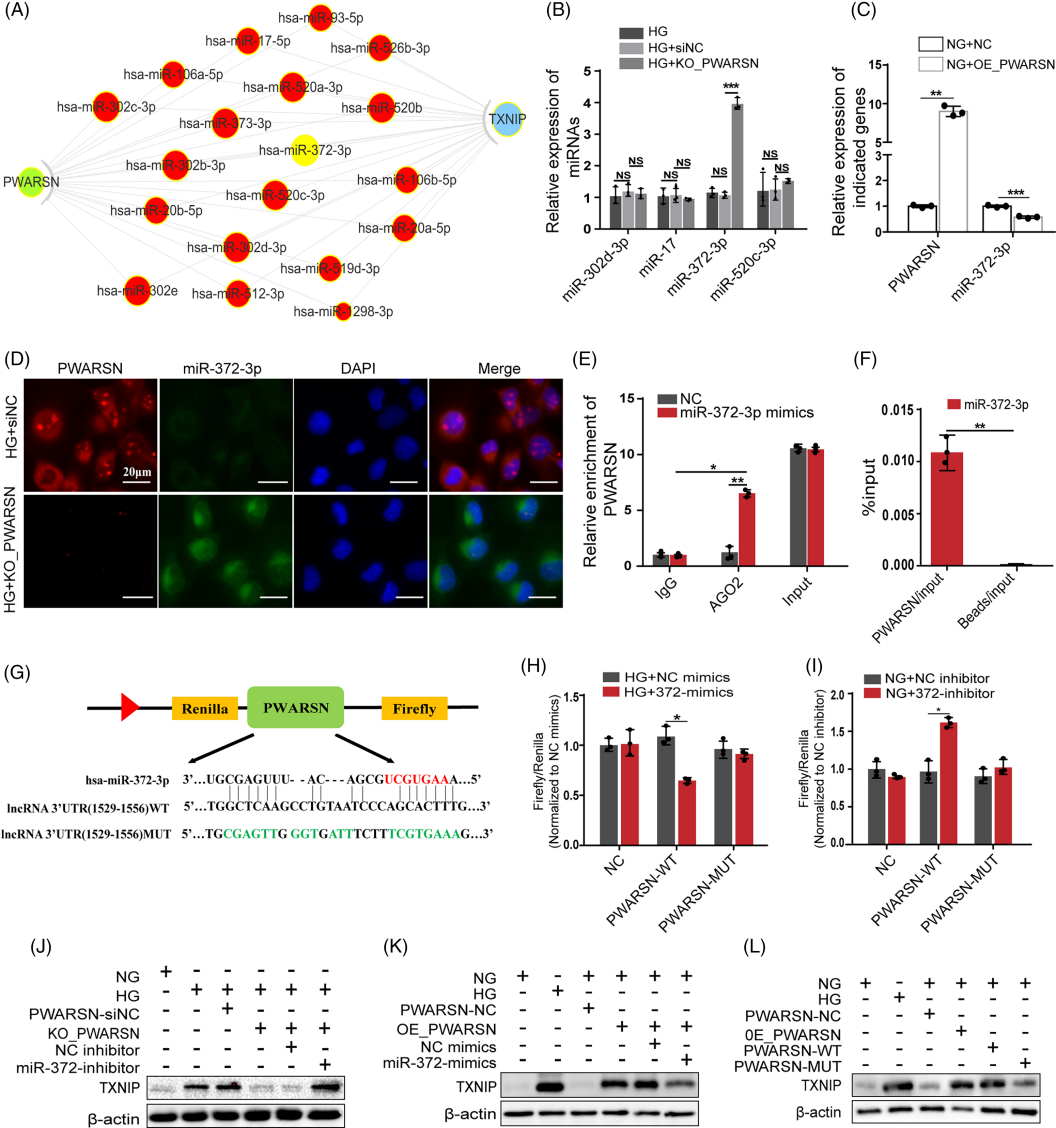
*PWARSN* sponges miR‐372‐3p in the cytoplasm to regulate TXNIP. (A) Competing endogenous RNA (ceRNA) network of *PWARSN‐* and *TXNIP*‐related miRNAs. (B) Relative expression of candidate miRNAs in *PWARSN*‐knockout HK‐2 cells. (C) Relative expression of *PWARSN* and miR‐372‐3p in *PWARSN*‐overexpressing HK‐2 cells. (D) FISH images showing the co‐localization of *PWARSN* (red) and miR‐372‐3p (green) in *PWARSN*‐knockout cells. Scale bar, 20 μm. (E) Anti‐AGO2 RIP assay was performed to detect *PWARSN* associated with AGO2. AGO2: Argonaute 2; RIP: RNA immunoprecipitation. (F) RNA interactome followed by qRT‐PCR showing the interaction between miR‐372‐3p and *PWARSN*. (G) Sequence of the wild‐type *PWARSN* 3′‐UTR and the mutant sequences on the complementary sites of *PWARSN* 3′‐UTR with miR‐372‐3p. (H‐I) Luciferase activity in HK‐2 cells co‐transfected with miR‐372‐3p mimics (H) and miR‐372‐3p inhibitor (I), and luciferase reporters containing empty plasmid, *PWARSN*, or mutant transcript. (J) The protein level of TXNIP in *PWARSN*‐knockout cells transfected with miR‐372‐3p inhibitor. (K) The protein level of TXNIP in *PWARSN*‐overexpressing cells transfected with miR‐372‐3p mimics. (L) Changes in protein level of TXNIP in *PWARSN*‐overexpressing cells transfected with the mutant plasmid of the 3′‐UTR *PWARSN* sequence for miR‐372‐3p. The data were obtained from at least three independent experiments. Data are presented as the means ± SD, and significance was determined using unpaired Student's *t*‐test (C, F, H, I) for two groups and one‐way ANOVA (B, E) for multiple groups (*p < 0.05; **p < 0.01; ***p < 0.001; NS, not significant).

To further investigate the detailed mechanism of *PWARSN's* regulation of TXNIP/NLRP3‐induced pyroptosis in DKD, we proposed the possibility that *PWARSN* acts as a competing endogenous RNA (ceRNA) for miRNA. We first established a ceRNA network and identified some *PWARSN*‐ and *TXNIP*‐related miRNAs (Figure 5A). Several miRNAs were downregulated in the HG group; however, miR‐372‐3p was upregulated in *PWARSN‐*knock out cells and was downregulated in *PWARSN‐*overexpressing cells (Figure 5B, C, and Figure S7A). FISH assay showed an increase in miR‐372‐3p after *PWARSN* was knocked out (Figure 5D). RNA immunoprecipitation ‐PCR (Figure 5E), RNA interactome (Figure 5F) and dual‐luciferase reporter assays confirmed the binding of miR‐372‐3p to *PWARSN* (Figure 5G–I). Next, the effects of miR‐372‐3p on TXNIP and PTEC pyroptosis were explored. Dual‐luciferase reporter assays confirmed the binding of miR‐372‐3p to TXNIP (Figure S7B–E). Western blotting assay showed that miR‐372‐3p regulated TXNIP and pyroptosis related proteins in HK‐2 cells (Figure S7F). Then, we found that *TXNIP* overexpression reversed the change in NLRP3 protein level mediated by miR‐372‐3p mimics, but transfecting TXNIP overexpressing plasmid harbouring the 3′‐ untranslated region mutant sequence had no effect (Figure S7G). Additionally, miR‐372‐3p inhibitor abolished the downregulation of TXNIP level induced by knocking out *PWARSN*, while miR‐372‐3p mimics abolished the upregulation of TXNIP level induced by overexpressing *PWARSN* (Figure 5J, K). Notably, overexpression of *PWARSN* increased the abundance of TXNIP and NLRP3, but the plasmid harbouring the 3′‐untranslated region mutant sequence of *PWARSN* that was bound to miR‐372‐3p did not affect the protein levels of TXNIP and NLRP3 (Figure 5L). Collectively, these findings suggest that *PWARSN* functioning is dependent on sponging cytoplasmic miR‐372‐3p partly.

### 3.5 Nuclear *PWARSN* promotes RBMX degradation via the ubiquitination‐proteasome pathway

**FIGURE 6:**
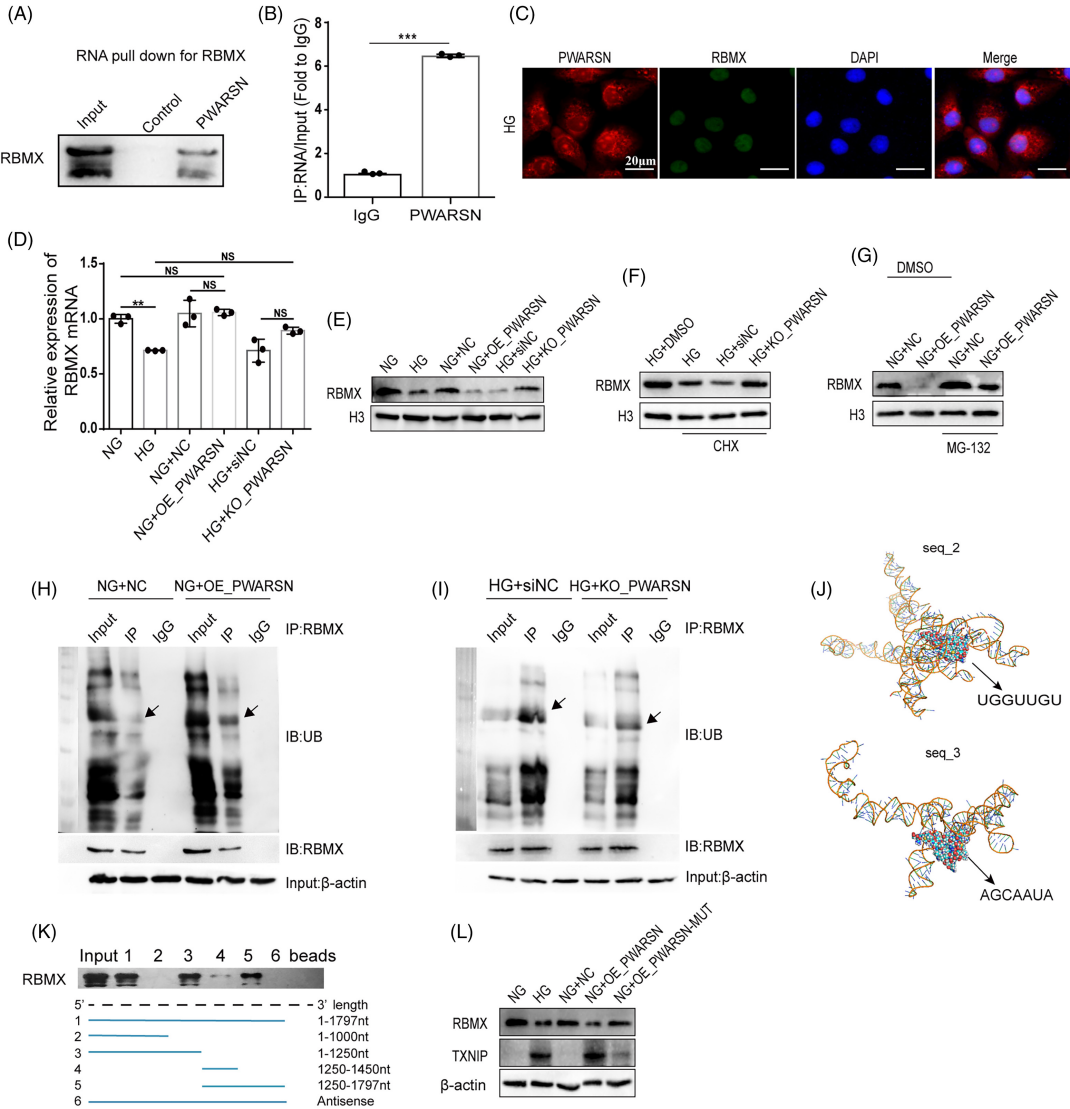
Nuclear *PWARSN* promotes RBMX degradation via the ubiquitination‐proteasome pathway. (A) The interaction between *PWARSN* and RBMX was confirmed by RNA pull‐down and western blotting. (B) *PWARSN* interaction with endogenous RBMX in HK‐2 cells was confirmed by RNA immunoprecipitation (RIP) analysis. (C) FISH images showing the co‐localization of *PWARSN* (red) and RBMX (green) in HK‐2 cells. Scale bar, 20 μm. (D–E) RBMX mRNA (D) and protein (E) levels in *PWARSN*‐overexpressing and *PWARSN*‐knockout HK‐2 cells by qRT‐PCR and western blotting. (F) The level of RBMX protein was detected in HG‐treated and *PWARSN*‐knockout HK‐2 cells incubated with the protein synthesis inhibitor cycloheximide (CHX, 100 μg/ml). (G) The level of RBMX protein was detected in NG‐treated and *PWARSN*‐overexpressing HK‐2 cells incubated with MG‐132 (10 nmol/ml). (H‐I) Cell lysates were immunoprecipitated with antibody against RBMX and analysed by immunoblotting with ubiquitin (Ub)‐specific antibody and anti‐RBMX antibody. Bottom: Input from cell lysates. (J) Schematic of the interaction between *PWARSN* and RBMX created using HNADOCK. The binding regions are shown in two different nucleotide sequences of *PWARSN*. (K) RNA pull down showing the interaction of sequentially deleted *PWARSN* mutants with RBMX in vitro. (L) The protein levels of RBMX and TXNIP in HK‐2 cells transfected with *PWARSN* and mutant plasmid of *PWARSN* interacting with RBMX. Data are presented as the means ± SD, and significance was determined using unpaired Student's *t*‐test (B) for two groups and one‐way ANOVA (D) for multiple groups (*p < 0.05; **p < 0.01; ***p < 0.001; NS, not significant).

Notably, *PWARSN* was accumulated in both the cytoplasm and nucleus. Given that lncRNAs exhibit their effects dependent on their localization in cells, we wonder about the function of nuclear *PWARSN*. To further identify the activity of nuclear *PWARSN*, we performed chromatin isolation via RNA purification followed by mass spectrometry analysis (Figure S8A–D). We failed to detect a direct interaction between *PWARSN* and TXNIP protein but discovered that RBMX, a protein that participates in the regulation of lncRNAs and TXNIP,^33^, ^34^ was one of the most abundant proteins (Figure S8E). An RNA pull‐down assay followed by western blotting, RNA immunoprecipitation (RIP) assays and FISH further confirmed the interaction and colocalization between RBMX and *PWARSN* (Figure 6A–C). *PWARSN* deficiency effectively increased the RBMX protein levels in HK‐2 cells but not the *RBMX* mRNA levels (Figure 6D, E). To elucidate the mechanism of RBMX dysregulation, the protein synthesis inhibitor cycloheximide was applied to HG‐treated cells. We found that cycloheximide (CHX) intervention significantly reduced RBMX protein levels, but had no effect in *PWARSN*‐knockout cells (Figure 6F). Moreover, treatment with the proteasome inhibitor MG‐132 enhanced RBMX levels in both NG‐treated and *PWARSN*‐overexpressing cells (Figure 6G). Importantly, *PWARSN* overexpression and knockout enhanced and reduced RBMX ubiquitination degradation in HK‐2 cells, respectively (Figure 6H, I), suggesting that protein degradation via ubiquitination‐proteasome is critical for the regulatory effect of *PWARSN* on RBMX.

To further determine the specific functional region(s) of *PWARSN* that are critical in the regulation of RBMX, catRAPID software (http://service.tartaglialab.com/page/ catrapid_group) was used to predict the enrichment of binding regions between *PWARSN* and RBMX protein (Figure S8F). Analysis by RNAfold software followed by HANDOCK software analysis showed the presence of several RBMX motifs in the *PWARSN* sequence (Figure 6J, and Figure S8G). Moreover, RNA pull‐down and truncation analyses suggested that fragments of 1000–1250 nt and 1450–1797 nt are critical for RBMX binding (Figure 6K). Corroborating these findings, *PWARSN* carrying mutations in both 1000–1250 nt and 1450–1797 nt fragments failed to downregulate RBMX protein level or upregulate TXNIP protein level (Figure 6L), indicating that *PWARSN*‐ regulated *TXNIP* expression is dependent on enhancing RBMX ubiquitin‐dependent degradation.

### 3.6 *PWARSN* epigenetically regulates TXNIP by interacting with RBMX

**FIGURE 7:**
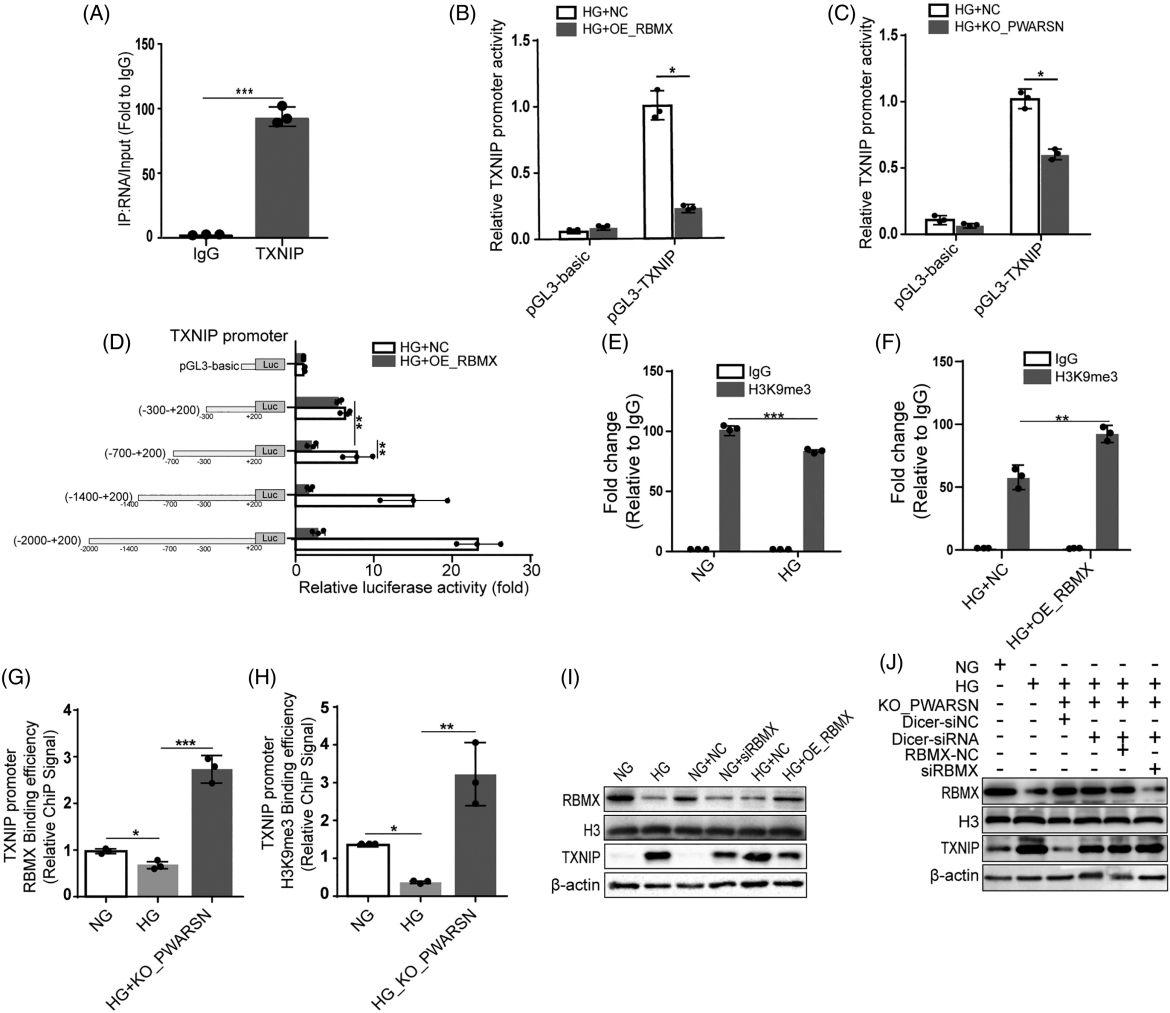
*PWARSN* epigenetically regulates TXNIP by interacting with RBMX. (A) RNA immunoprecipitation (RIP) analysis using the anti‐RBMX antibody revealed that TXNIP mRNA interacted with endogenous RBMX in HK‐2 cells. (B and C) Luciferase reporter gene activity linked to the TXNIP promoter was decreased in RBMX‐overexpressing (B) and *PWARSN*‐knockout (C) HK‐2 cells. (D) Transcriptional activity was evaluated using sequential deletions and by examining the TXNIP promoter linked to renilla luciferase activity in RBMX‐overexpressing HK‐2 cells. (E and F) The enrichment of H3K9me3 at TXNIP promoter was evaluated using ChIP assay in NG, HG (E) and RBMX‐overexpressing HK‐2 cells (F). (G and H) The enrichments of RBMX (G) and H3K9me3 (H) at TXNIP promoter were evaluated using ChIP assay in NG‐treated, HG‐treated and *PWARSN*‐knockout HK‐2 cells. (I) The protein levels of TXNIP and RBMX in RBMX‐overexpressing and RBMX‐knockdown HK‐2 cells. (J) Knocking down RBMX abolished the reduction of TXNIP level and upregulation of RBMX level in *PWARSN‐*knockout HK‐2 cells by western blotting. The data were obtained from at least three independent experiments. Data are presented as the means ± SD, and significance was determined using unpaired Student's *t*‐test (A–C, E, F) for two groups and one‐way ANOVA (D, G, H) for multiple groups (*p < 0.05; **p < 0.01; ***p < 0.001).

Considering that RBMX is a transcription factor with abundant binding regions on TXNIP as confirmed by RIP‐qPCR (Figure 7A), we performed luciferase reporter assays and found that both *RBMX* overexpression and *PWARSN* deficiency reduced the luciferase activity of the *TXNIP* promoter driven by RBMX (Figure 7B, C). Then, we cloned a series of TXNIP‐ luciferase promoter constructs, ranging from −2000 nt to +200 nt relative to the transcriptional start site to explore the specific functional regions. The results revealed a clear reduction in the transcriptional activity of the construct from −700 to −300 bp (Figure 7D). A previous study reported that RBMX recruits H3K9me3 to regulate gene silencing.^35^ Chromatin immunoprecipitation assays in our study revealed that HG decreased the enrichment of H3K9me3 at the *TXNIP* promoter (Figure 7E); however, *RBMX* overexpression in HG‐treated cells increased the enrichment of H3K9me3 (Figure 7F). Furthermore, we found that HG decreased the enrichment of RBMX and H3K9me3 on the *TXNIP* promoter; however, *PWARSN* knockout enhanced the enrichment (Figure 7G, H). In support of these findings, *RBMX* overexpression downregulated TXNIP level in HG‐treated cells whereas *RBMX* knockdown led to the opposite effects (Figure 7I). Additionally, we used small interference RNA of Dicer (ribonuclease III) to efficiently block miRNA biogenesis in HK‐2 cells and found that *PWARSN*‐knockout failed to reduce TXNIP level in *RBMX*‐silenced cells (Figure 7J, and Figure S8H). Notably, no remarkable interaction was observed between RBMX and miR‐372‐3p (Figure S8I, J). Collectively, we propose that *PWARSN* may act as a decoy and titrate away RBMX and its partners from the *TXNIP* promoter, resulting in H3K9me3 being diminished and *TXNIP* transcription being activated.

### 3.7 *PWARSN* can be used as a biomarker for DKD

**FIGURE 8:**
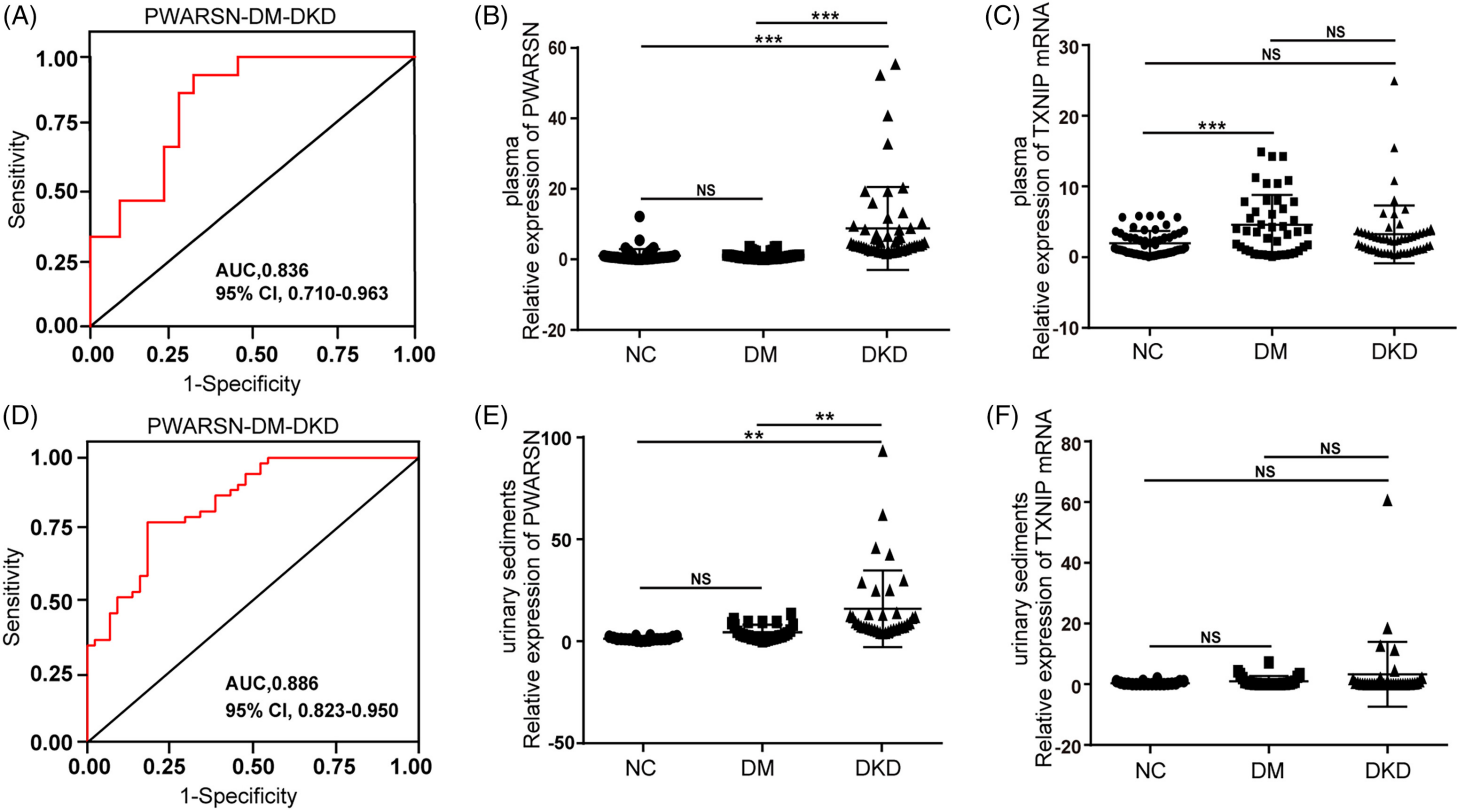
*PWARSN* can be used as a biomarker for DKD. (A) Receiver operating characteristic curve (ROC) analysis of the power of *PWARSN* in the renal samples from patients with DM and DKD. AUC: area under the curve; CI: confidence interval. (B and C) Relative *PWARSN* (B) and TXNIP mRNA(C) levels in plasma samples from healthy individuals with NGT (NC, *n* = 56), patients with DM (DM, *n* = 44), and patients with DKD (DKD, *n* = 53). NGT: normal glucose tolerance; DM: diabetes mellitus; DKD: diabetic kidney disease. (D) Receiver operating characteristic curve (ROC) analysis of the power of *PWARSN* in plasma samples from patients with DM and DKD. AUC, area under the curve; CI, confidence interval. (E and F) Relative *PWARSN* (E) and TXNIP mRNA (F) levels in urinary sediment samples from healthy individuals with NGT (NC, *n* = 20), patients with DM (DM, *n* = 28), and patients with DKD (DKD, *n* = 36). Data are presented as the means ± SD, and significance was determined using Kruskal‐Wallis Test (B, C, E, F) for multiple groups (*p < 0.05; **p < 0.01; ***p < 0.001; NS: not significant).

Considering the important role of *PWARSN* in the pathogenesis of DKD, a diagnostic model of DKD based on *PWARSN* expression level was established. We first focused on the expression level of *PWARSN* in renal tissue collected from previous 69 patients. The sensitivity and specificity of *PWARSN* as a biomarker of DKD were 93.33% and 68.18%, respectively, with a receiver operating characteristic (ROC) area of 0.836 (Figure 8A). Considering the inconvenience of performing a renal biopsy, we tested the levels of *PWARSN* and TXNIP mRNA in 153 human plasma samples obtained from 56 healthy kidneys of individuals with NGT, 44 patients with DM, and 53 patients with DKD (Table S3). The expression level of *PWARSN* was elevated in the plasma samples from the patients with DKD compared with that in the patients with DM (Figure 8B), whereas there was no significant difference in the level of TXNIP mRNA. However, the level of TXNIP mRNA was elevated in the patients with DM compared with that in the healthy individuals (Figure 8C). The sensitivity and specificity of *PWARSN* as a biomarker of DKD in the plasma samples were 85.19% and 81.82%, respectively, with an ROC area of 0.886 (Figure 8D). Furthermore, we investigated the levels of *PWARSN* and TXNIP mRNA in human urinary sediment samples obtained from 20 healthy individuals with NGT, 28 patients with DM and 36 patients with DKD (Table S4). Although the *PWARSN* level was significantly higher in the urinary sediment samples obtained from patients with DKD than in the samples obtained from patients with DM (Figure 8E), there was no significant difference in the level of TXNIP mRNA between the three groups (Figure 8F). Additionally, the sensitivity (72.22%) and specificity (64.29%) of *PWARSN* as a biomarker of DKD in urinary sediment samples were low, with an ROC area of only 0.751 (Figure S9).

## 4 DISCUSSION

Studies have confirmed that TXNIP is a central oxidative stress regulator that triggers inflammation as well as cell pyroptosis in DKD.^17^ Thus, clarifying the underlying mechanisms of TXNIP dysregulation contributes to exploring the therapeutic targets of DKD. Here, we identified a novel PTEC‐enriched lncRNA, *PWARSN*, which is a key regulator that specifically targets *TXNIP* to initiate PTEC pyroptosis in DKD. Mechanistically, cytoplasmic *PWARSN* sponges miR‐372‐3p to maintain TXNIP mRNA level. Moreover, nuclear *PWARSN* physically facilitates the degradation of RBMX protein via the ubiquitination‐proteasome pathway, thereby reducing H3K9me3 at the *TXNIP* promoter and promoting *TXNIP* transcription. Defining this dual role for *PWARSN* leads to a deeper understanding of the cross‐talk between *PWARSN*, TXNIP, pyroptosis and DKD, highlighting the role of *PWARSN* which might be a novel potential biomarker.

Pyroptosis is characterized by rapid cell membrane rupture and the release of proinflammatory contents, which disrupts ion homeostasis and ultimately causes cell death.^36^ Studies have indicated the pivotal role of ROS‐induced pyroptosis in regulating many inflammatory disorders.^37^, ^38^ Increased PTEC pyroptosis contributes to the pathology of acute kidney injury.^39^ PTEC pyroptosis mediated by Gasdermin E activation results in inflammation and fibrosis in obstructive nephropathy.^40^ Caspase‐11/4‐Gasdermin D activation contributes to podocyte pyroptosis in diabetic mice.^13^ In this study, we observed that PTEC pyroptosis was activated in the patients, cell cultures, and in the mouse model of DKD. Considering the great promoting role of TXNIP in ROS production, TXNIP was able to induce NLRP3 inflammasome activation and PTEC pyroptosis in DKD.^11^ Pyroptosis is not only a normal way to induce cell death but also an amplifier of excessive inflammatory injury to the renal tubules, which may explain why the inflammatory response leads to rapid renal function deterioration in patients with DKD.

LncRNAs play a critical role in cellular processes and the alteration in their expression is well‐connected to the progression of various diseases.^41^ Several dysregulated lncRNAs have been functionally validated as important regulators in DKD.^42^, ^43^ However, very few studies have demonstrated the regulatory function of lncRNAs in cell pyroptosis in the renal tubules of DKD. In this study, we found that lncRNA‐ *PWARSN* is a key driver of TXNIP and has the capability to regulate PTEC pyroptosis in DKD. Studies have demonstrated that TXNIP‐derived ROS can induce NLRP3 inflammasome activation, and TXNIP/ROS oxidase signaling is also involved in the development of DKD. In addition, the inhibition of TXNIP‐responsive oxidative stress and the ROS‐sensitive NLRP3 signaling pathway protects against diabetic kidney injury.^44^ Our data showed that *PWARSN* increased cellular ROS generation and TXNIP, NLRP3 inflammasomes activation, leading to PTECs pyroptosis both in vitro and in vivo. However, knocking out *PWARSN* in PTECs with the CRISPR‐Cas9 system improved intracellular redox reaction dysregulation and prevented the TXNIP increase, accompanied by the amelioration of cell pyroptosis.

Currently, less than 5% of human lncRNAs have been functionally identified between species and most lncRNAs are poorly conserved.^45^, ^46^ Given that *PWARSN* could not be identified in rats or mice, we could not construct a renal tubular *PWARSN*‐specific knockout mouse model. However, researchers have explored many methods to investigate the functions of non‐conserved human lncRNAs in different species. For example, researchers transplanted cells expressing primate‐specific lncRNA in rodent models with immune deficiency to explore the role of human lncRNAs in tumour metastasis.^47^ Wang et al injected non‐conserved lncRNA into the kidney of a mouse model to improve renal interstitial fibrosis.^48^ Ruan et al also identified the role of non‐conserved lncRNAs in cholesterol metabolism through humanized mouse model and conventional ectopic expression in mice.^49^ These data suggest that non‐conserved lncRNAs are unique and indispensable parts of human genetic regulatory networks. Thus, we transfected human *PWARSN* plasmid into mRTECs and AAV9‐*PWARSN* virus into mouse kidneys according to methods used in previous studies and found that *PWARSN* could induce the upregulation of ROS level and mRTEC pyroptosis, leading to deteriorated renal dysfunction, which may be related to conserved spatial structure,^50^ transcriptional position,^51^ splicing process^52^ and conserved functions.^53^, ^54^ Overall, our results suggested that *PWARSN* constitutes a conserved, finely regulated mechanism during DKD development.

LncRNAs exhibit their effects through epigenetic modification, protein stability, gene transcription, splicing and trafficking, which depend on their cellular localization.^18^ Likewise, the newly identified *PWARSN* regulated the expression of *TXNIP*; however, its regulatory mechanism was different from that of other lncRNAs. We found that *PWARSN* was distributed in the cytoplasm and nucleus. Initially, our data indicated that cytoplasmic *PWARSN* sponged miR‐372‐3p to regulate TXNIP‐induced PTEC pyroptosis. Then we found that targeting miR‐372‐3p may only partially antagonize the regulatory effect of *PWARSN* on TXNIP, which was reminiscent of the unknown biological effect of nuclear *PWARSN*. Thus, we screened for the proteins that may bind *PWARSN* and mediate its effects. The RNA pull‐down and RIP assays reciprocally proved that RBMX is a direct binding protein of *PWARSN* in the nucleus. We further confirmed that the regulatory effect of *PWARSN* on TXNIP was mediated by RBMX. As introduced before, there are many pathways and complex feedback systems to regulate the expression of TXNIP and ROS generation.^16^, ^44^ As an important stress sensor, TXNIP interplays with many pyroptosis regulatory pathways.^55^, ^56^ Extra TXNIP increased progressively with DKD in diabetic kidneys and is responsible for tubular oxidative injury.^57^ In the present study, our results suggested that not only cytoplasmic *PWARSN* sponged miR‐372‐3p to promote TXNIP expression, but also nuclear *PWARSN* interacted and facilitated RBMX degradation to initiate TXNIP transcription, resulting in the activation of NLRP3 inflammasome and cell pyroptosis. This dual role provides the first and most comprehensive evidence that *PWARSN* dysregulation is the key factor that catalyses the imbalance of TXNIP in renal tubules in DKD, and this contributes to the exploration of the target for DKD treatment.

Interestingly, our current study demonstrated that *PWARSN* was highly expressed in both renal tubules, plasma and urinary sediment samples collected from patients with DKD. We found that the plasma specimens and renal tubule samples showed high sensitivity, specificity, and notable ROC areas after further establishing diagnostic models based on our results. However, the results of the urine sediment samples were unsatisfactory, which might be due to the limited number of samples. Thus, the clinical significance of *PWARSN* in the diagnosis of DKD needs to be verified in larger clinical studies. Notably, although our data showed that the expression of TXNIP mRNA was increased in renal tissues from patients with DKD, there were no significant differences in plasma and urinary sediment samples compared to those from patients with DM. Further detailed studies are warranted to address this phenomenon.

In conclusion, these findings demonstrate that *PWARSN* is an important driver of DKD progression, aggravating PTEC pyroptosis by activating the TXNIP/NLRP3 signaling pathway via a dual mechanism. These findings pave the way for *PWARSN* to serve as a potential diagnostic biomarker and a therapeutic target for DKD in the future.

## AUTHOR CONTRIBUTIONS

Yi Song, Feng Guo, Qing‐zhu Wang and Gui‐jun Qin designed the research. Yi Song and Feng Guo performed most of the experiments. Yan‐yan Zhao, Xiao‐jun Ma, Li‐na Wu, Hong‐fei Ji and Ming‐wei Shao participated in the experiments and data analysis. Feng‐juan Huang and Lin Zhao handled the kidney, plasma, and urinary sediment samples. Xun‐jie Fan and Ya‐nan Xu analysed kidney, plasma, and urinary sediment samples. Yi Song and Feng Guo wrote the manuscript with assistance from Ji‐feng Yu, Qing‐zhu Wang, Gui‐jun Qin and Qing‐zhu Wang. Ji‐feng Yu and Feng Guo edited the manuscript. All authors read and approved the final manuscript.

## CONFLICT OF INTEREST

The authors declare that they have no conflict of interest.

## Supporting information


**Appendix S1.** Supporting Information.

## Data Availability

The authors declare that all data supporting the findings of this study are available within the article or are available from the corresponding author upon request.
