# Supplementary material for: Novel lncRNA‐prader willi/angelman region RNA, SNRPN neighbour (PWARSN) aggravates tubular epithelial cell pyroptosis by regulating TXNIP via dual way in diabetic kidney disease
Source: Cell Prolif. 2022 Oct 31;56(2):e13349. doi: 10.1111/cpr.13349 (PMC9890532; doi:10.1111/cpr.13349)
Supplement: Supplementary file 1 — Appendix S1. Supporting Information. [file CPR-56-e13349-s001.docx]

**Supplemental material Table of Contents**

**Figure S1.** TXNIP and NLRP3 inflammasome were activated in kidneys of diabetic mice

**Figure S2.** TXNIP induces NLRP3 inflammasome activation and proximal tubular epithelial cells pyroptosis under high glucose condition

**Figure S3.** *PWARSN* is specially and highly expressed in PTECs

**Figure S4.** *PWARSN* triggers TXNIP/NLRP3-related pyroptosis of tubular epithelial cells under high glucose condition

**Figure S5.** Ectopic expression of human *PWARSN* induces mRTECs pyroptosis

**Figure S6.** Delivery of human *PWARSN* contributes to tubular inflammation and mRTECs pyroptosis in wild-type mice

**Figure S7.** MiR-372-3p regulates TXNIP

**Figure S8.** *PWARSN* interacts with RBMX

**Figure S9.** The ROC analysis of the power of *PWARSN* in urinary sediment samples from patients with DM and DKD

**Table S1**. Clinical characteristics of patients in pathological samples

**Table S2.** Body weight, kidney weight/body weight ratio and biochemical indicators in normal (NC), diabetic (DM) and saline/Vector/*PWARSN* groups

**Table S3.** Clinical characteristics of patients in plasma samples

**Table S4.** Clinical characteristics of patients in urine sediments samples

**Table S5.** siRNA oligos and primers

**Table S6.** The list of lncRNAs in lncRNA-TXNIP co-expression network

**Supplementary Figures**

**Figure S1**


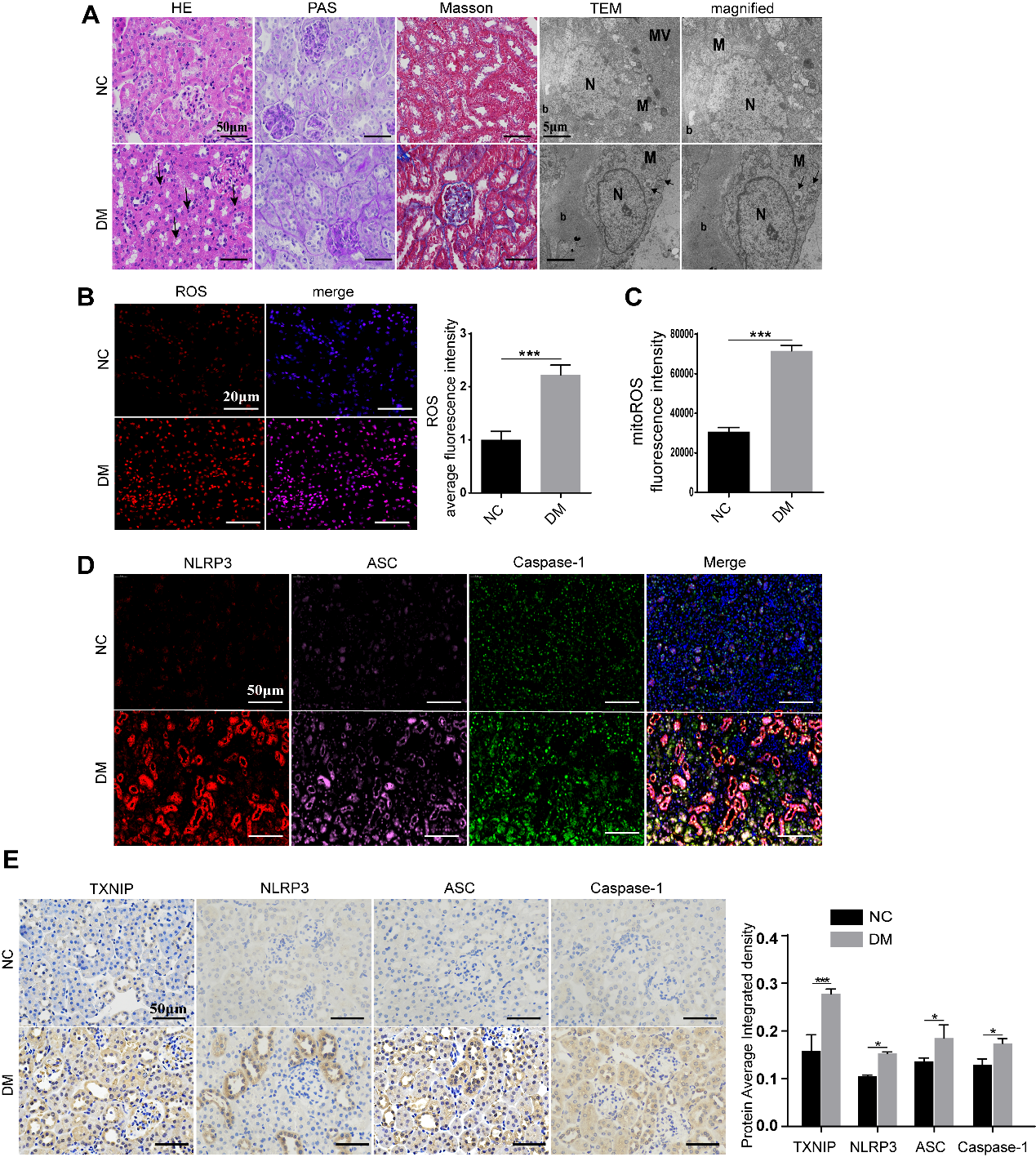


**Figure S1 TXNIP and NLRP3 inflammasome were activated in kidneys of diabetic mice**

(**A-E)** Kidney samples were obtained from normal and diabetic mice. NC: normal control mice; DM: diabetic mice. (**A**) Representative images viewed using HE (black arrows: inflammatory cells), PAS, Masson’s staining and TEM (DM: thickened basement membrane, disrupted microvilli, few mitochondria with hardly recognizable structures, and autophagic vacuoles. b: basement membrane; N: nucleus; M: mitochondria; MV: microvilli; black arrows: autophagic vacuoles). HE: hematoxylin and eosin; PAS: periodic-acid schiff; TEM: transmission electron microscopy. Scale bar, 50 µm, 5 µm. **(B-C)** Intracellular ROS (**B**) and mitochondrial ROS (**C**) levels of renal samples in mice were assessed using immunofluorescence microscopy. Scale bar, 20 µm. **(D)** Co-localization of Nlrp3 (red), Asc (pink) and caspase-1 (green) viewed using scanning immunofluorescence microscopy. Scale bar, 50 µm. **(E)** Immunohistochemical staining of kidneys for Txnip, Nlrp3, Asc and caspase-1 in diabetic mice. *n* = 3 per group. Scale bar, 50 µm. Data are presented as the means ± SD, and significance was determined using unpaired Student’s *t*-test (**B, C**) for two groups and one-way ANOVA (**E**) for multiple groups. (**P* <0.05; ****P* <0.001).

**Figure S2**


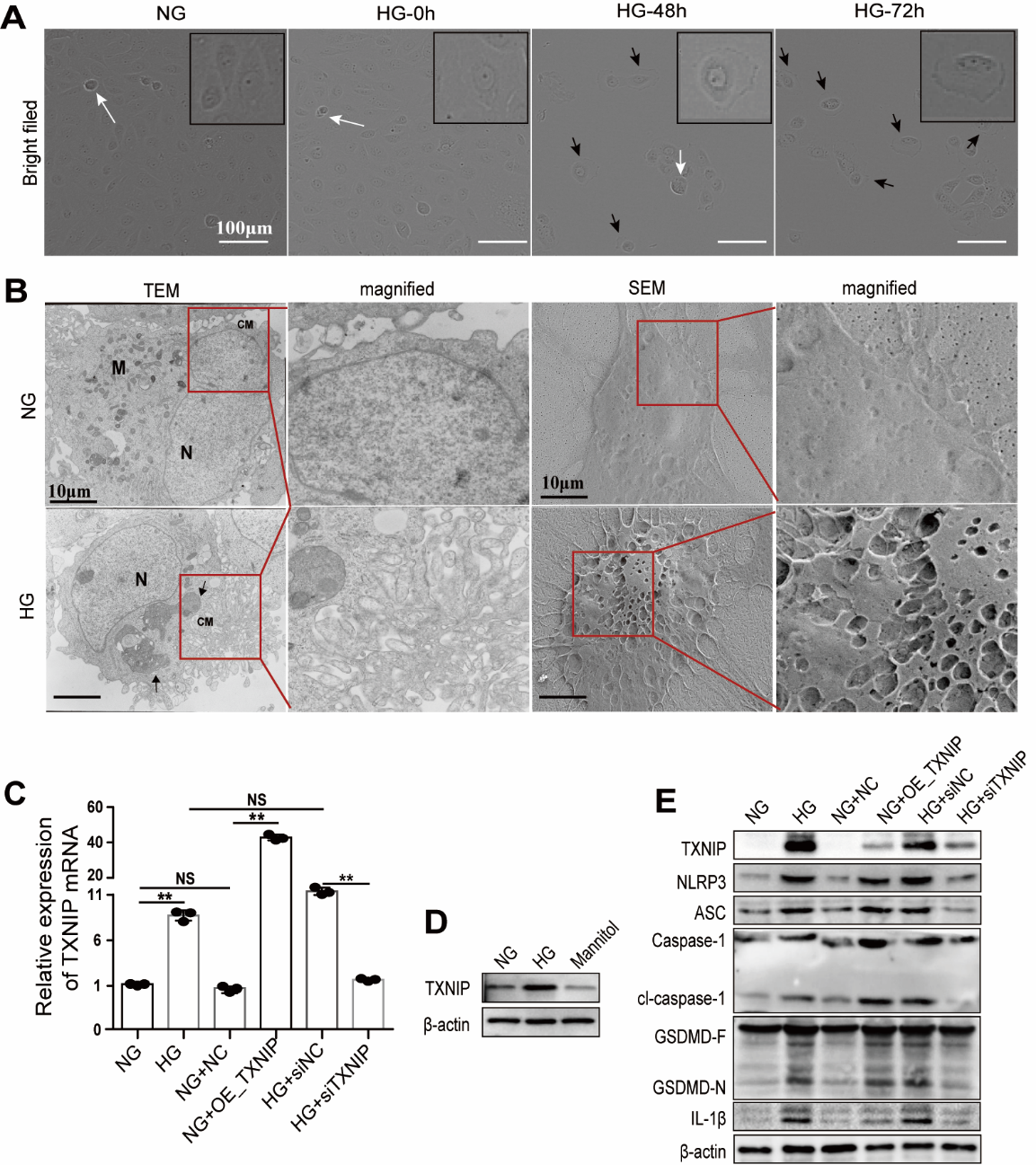


**Figure S2** **TXNIP induces NLRP3 inflammasome activation and proximal tubular epithelial cells pyroptosis under high glucose condition**

**(A)** Representative images of pyroptosis in dynamic live HK-2 cells treated with NG (5.6 mM) and HG (30 mM) viewed using IncuCyte assays. HG-treated cells showed typical characteristics of pyroptosis such as swelling, bubbling and rupturing. white arrows: apoptotic cells; black arrows: pyroptotic cells. Scale bar, 100 µm. **(B)** Representative images of cell pyroptosis after NG and HG treatment viewed using TEM and SEM. (HG-treated cells showed increased mitochondria with hardly recognizable structures (M), increased autophagosome (black arrows) and dissolved cell membrane (CM)). SEM: scanning electron microscopy. Scale bar, 10 µm. **(C)** Relative TXNIP mRNA level in HK-2 cells transfected with TXNIP-overexpressing plasmid and TXNIP siRNA by qRT-PCR. **(D)** The protein level of TXNIP were not affected by mannitol (5.6 mM glucose + 24.4 mM mannitol) treatment. **(E)** The levels of pyroptosis-associated proteins in HK-2 cells transfected with TXNIP-overexpressing plasmid and TXNIP siRNA determined by western blotting. Data are presented as the means ± SD, and significance was determined using one-way ANOVA (**C**). (***P* <0.01, NS: not significant).

**Figure S3**


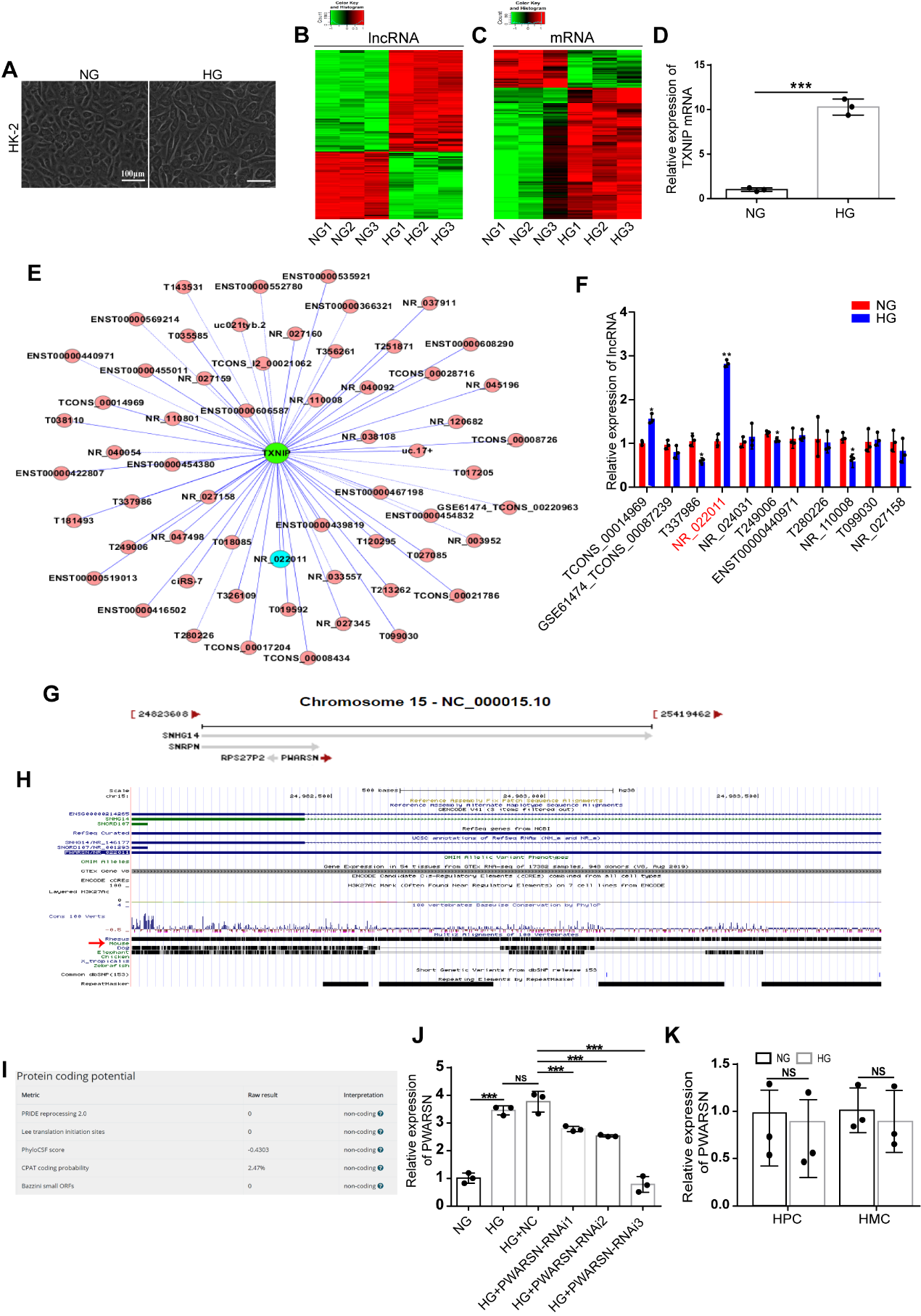


**Figure S3 *PWARSN* is specially and highly expressed in PTECs**

**(A)** Representative images of NG- and HG-treated HK-2 cells for 72 h. Scale bar, 100 µm. **(B-C)** Heatmaps of changes (fold change ≥ 2.0, *P* < 0.05) in the expression of lncRNAs **(B)** and mRNAs **(C)** in NG- and HG-treated HK-2 cells for 72 h. **(D)** Relative TXNIP mRNA level in NG- and HG-treated HK-2 cells by qRT-PCR. **(E)** LncRNA-TXNIP co-expression network in HK-2 cells. **(F)** Relative expression of the candidate lncRNAs in NG- and HG-treated HK-2 cells by qRT-PCR. **(G)** *PWARSN* is located at chromosome 15. **(H)** The conservation of *PWARSN* in the UCSC database. (Red arrow: There is no homologous sequence of *PWARSN* in mice) **(I)** *PWARSN* has no protein-coding potential as predicted using PhyloCSF and CPAT. **(J)** Relative expression of *PWARSN* in HK-2 cells transfected with mixture of siRNAs and antisense oligonucleotides for *PWARSN*. **(K)** Relative expression of *PWARSN* in HPC and HMC after NG and HG treatment for 72 h. HPC: human podocytes; HMC: human mesangial cells. Data are presented as the means ± SD, and significance was determined using unpaired Student’s *t*-test (**D, F, K**) for two groups and one-way ANOVA (**J**) for multiple groups. (**P* <0.05; ***P* <0.01; ****P* <0.001; NS: not significant).

**Figure S4**


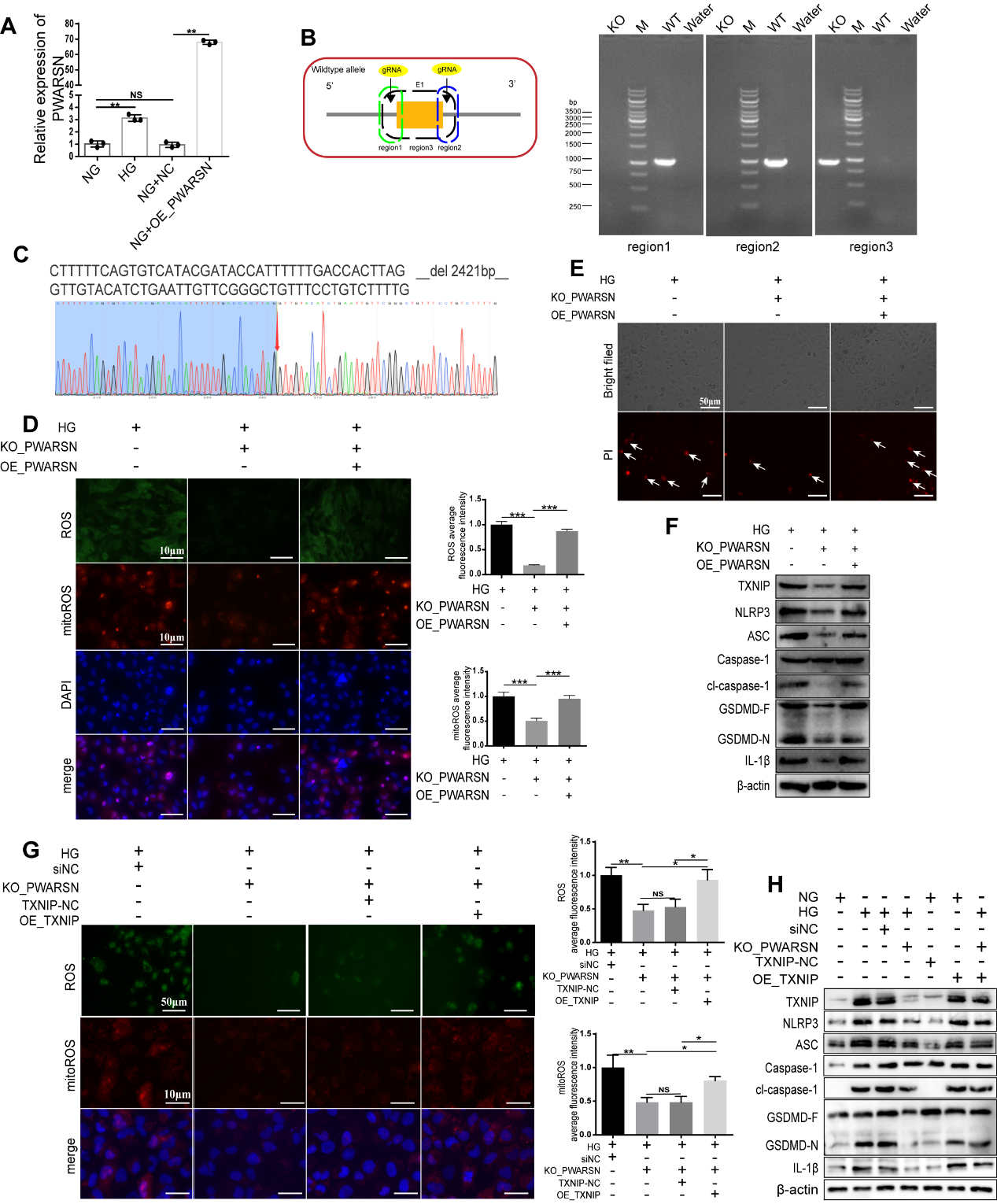


**Figure S4** ***PWARSN* triggers TXNIP/NLRP3-related pyroptosis of tubular epithelial cells under high glucose condition**

**(A)** Relative expression of *PWARSN* in HK-2 cells transfected with *PWARSN* using lentivirus system. **(B-C)** PCR **(B)** and gene sequencing **(C)** were used to confirmed the knockout of *PWARSN* in HK-2 cell. **(D)** Representative images of ROS and mitoROS in *PWARSN-* knockout HK-2 cells co-transfected with *PWARSN* overexpressing plasmid viewed using immunofluorescence microscopy. Scale bar, 10 µm. **(E)** Representative images of pyroptosis (red) in HK-2 cells assessed using propidium iodide staining. white arrows: pyroptotic cells. Scale bar, 50 µm. **(F)** The levels of TXNIP and pyroptosis-related proteins in *PWARSN*-knockout HK-2 cells co-transfected with *PWARSN*-overexpressing plasmid. **(G)** Representative images of ROS and mitoROS in *PWARSN-* knockout HK-2 cells co-transfected with *TXNIP* overexpressing plasmid viewed using immunofluorescence microscopy. Scale bar, 10 µm, 50 µm. **(H)** The levels of TXNIP and pyroptosis-related proteins in *PWARSN*-knockout HK-2 cells co-transfected with TXNIP-overexpressing plasmid. Data are presented as the means ± SD, and significance was determined using one-way ANOVA (**A, D**) for multiple groups. (**P* <0.05; ***P* <0.01; ****P* <0.001; NS: not significant).

**Figure S5**


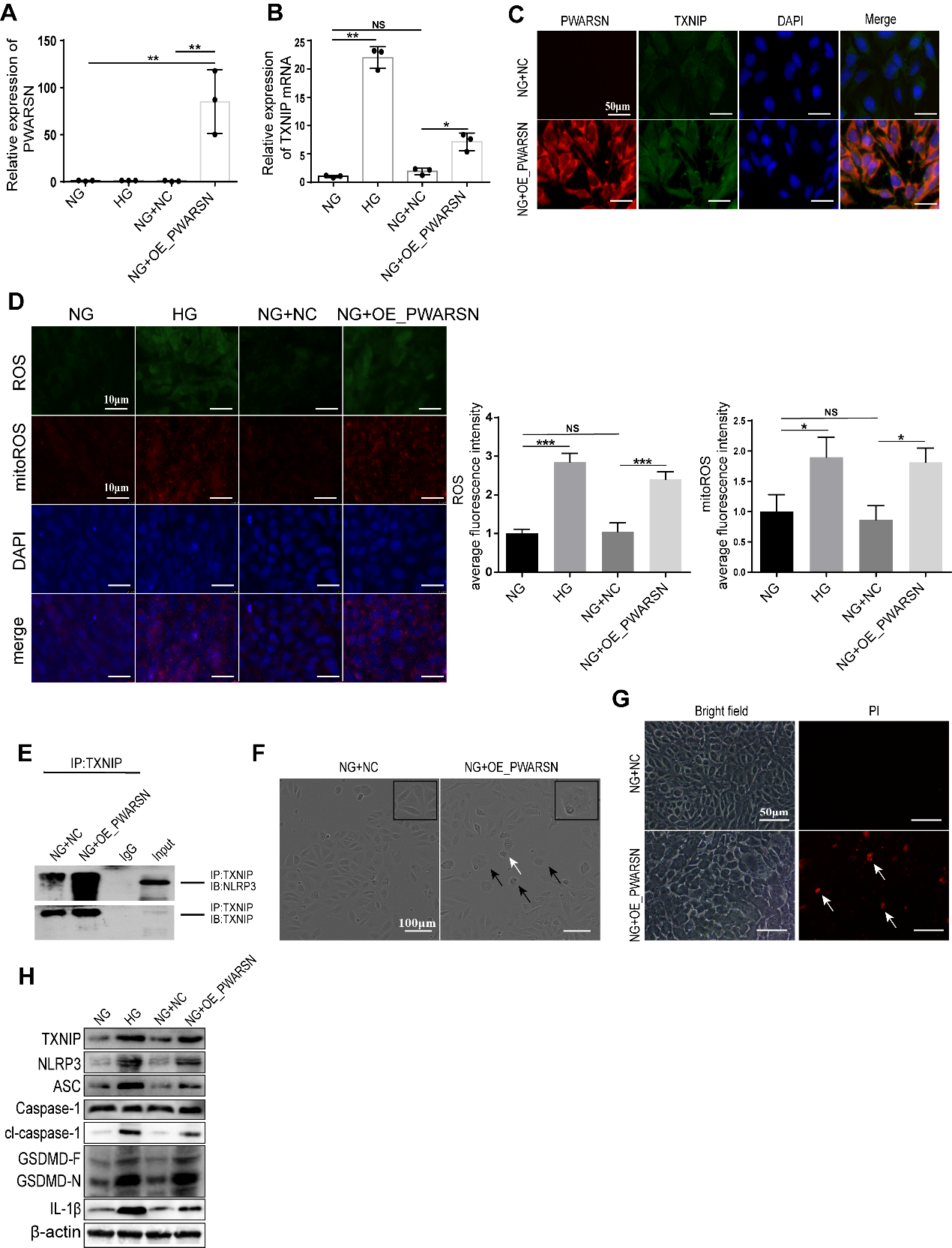


**Figure S5** **Ectopic expression of human *PWARSN* induces mRTECs pyroptosis**

**(A-B)** Relative *PWARSN* **(A)** and TXNIP mRNA **(B)** levels in human *PWARSN-*overexpressing mRTECs. **(C)** FISH assay showing the co-localization of human *PWARSN* (red) and TXNIP (green) in *PWARSN-*overexpressing mRTECs. Scale bar, 50 µm. **(D)** Transfection of human *PWARSN* increased the intracellular ROS and mitochondrial ROS levels in mRTECs. Scale bar, 10 µm. **(E)** Transfection of human *PWARSN* increased the interaction between TXNIP and NLRP3 in mRTECs using co-immunoprecipitation assay. **(F)** Representative images of pyroptosis in dynamic live human *PWARSN-*overexpressing mRTECs using IncuCyte assays. mRTECs showed typical characteristics of pyroptosis such as swelling, bubbling and rupturing. white arrows: apoptosic cell; black arrows: pyroptotic cells. Scale bar, 100 µm. **(G)** Cell pyroptosis (red) was assessed using propidium iodide staining in human *PWARSN-*overexpressing mRTECs. white arrows: pyroptotic cells. Scale bar, 50 µm. **(H)** The levels of Txnip and pyroptosis-related proteins in human *PWARSN-*overexpressing mRTECs. Data are presented as the means ± SD, and significance was determined using one-way ANOVA (**A, B, D**) for multiple groups. (**P* <0.05; ***P* <0.01; NS: not significant).

**Figure S6**


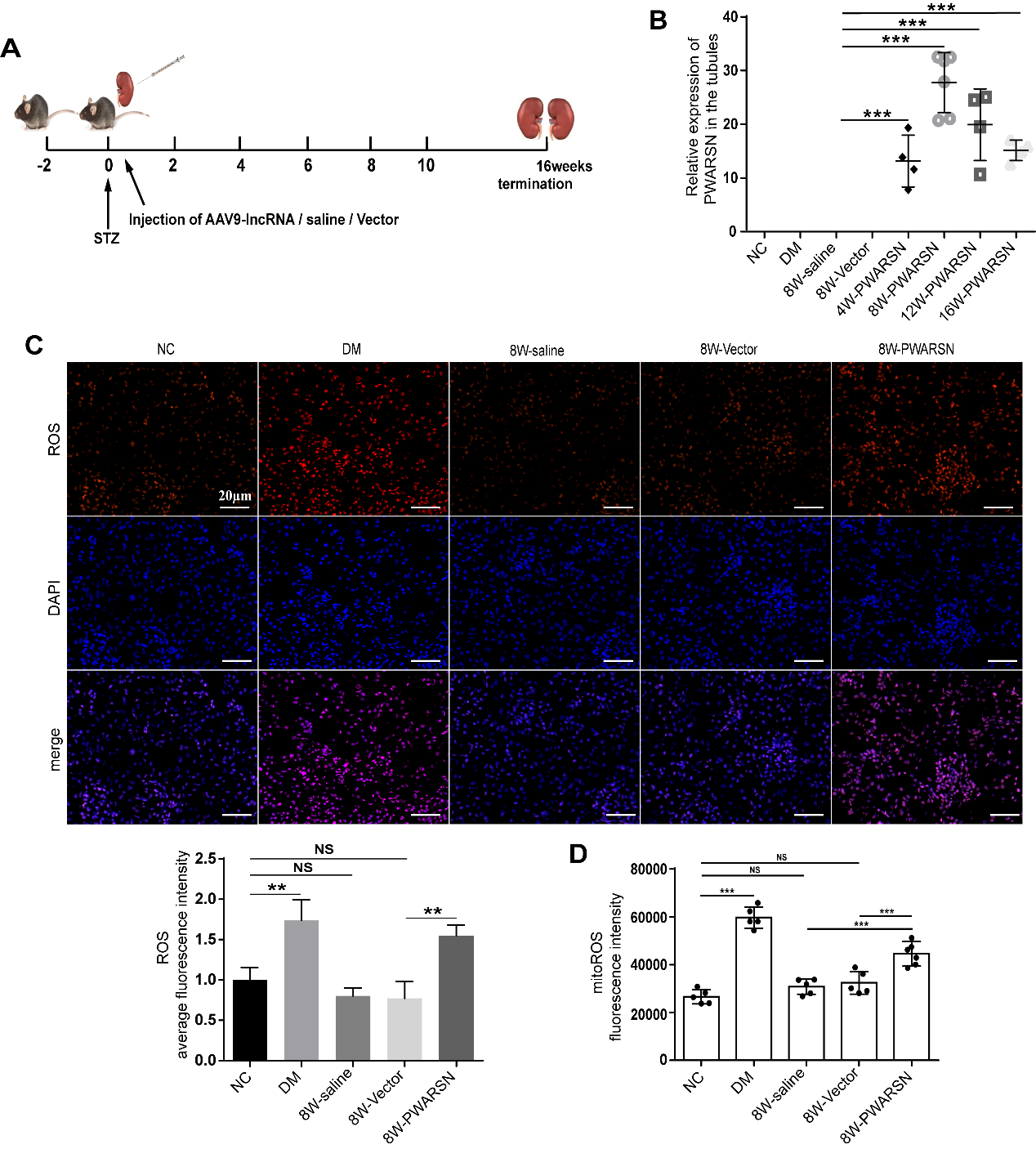


**Figure S6** **Delivery of human *PWARSN* contributes to tubular inflammation and mRTECs pyroptosis in wild-type mice**

**(A)** The renal cortexes of normal mice were injected with AAV9-*PWARSN*, saline and empty vector (Vector) at different weeks, and diabetic mouse was established by STZ. STZ: Streptozocin. **(B)** Relative expression of *PWARSN* in renal tubules of mice after injection with AAV9-*PWARSN* at different time points. NC, *n* = 5; DM, *n* = 5; 8W-saline, *n* = 5; 8W-Vector, *n* = 5; 4W-*PWARSN*, *n* = 4; 8W-*PWARSN*, *n* = 6; 12W-*PWARSN*, *n* = 4;16W-*PWARSN*, *n* = 4. NC: normal control mice; DM: diabetic mice. (**C-D)** Intracellular ROS (**C**) and mitochondrial ROS (**D**) levels of renal samples in mice were assessed using immunofluorescence microscopy. Scale bar, 20 µm. Data are presented as the means ± SD, and significance was determined using one-way ANOVA (**B, C, D**) for multiple groups. (**P* <0.05; ***P* <0.01; ****P* <0.001; NS: not significant).

**Figure S7**


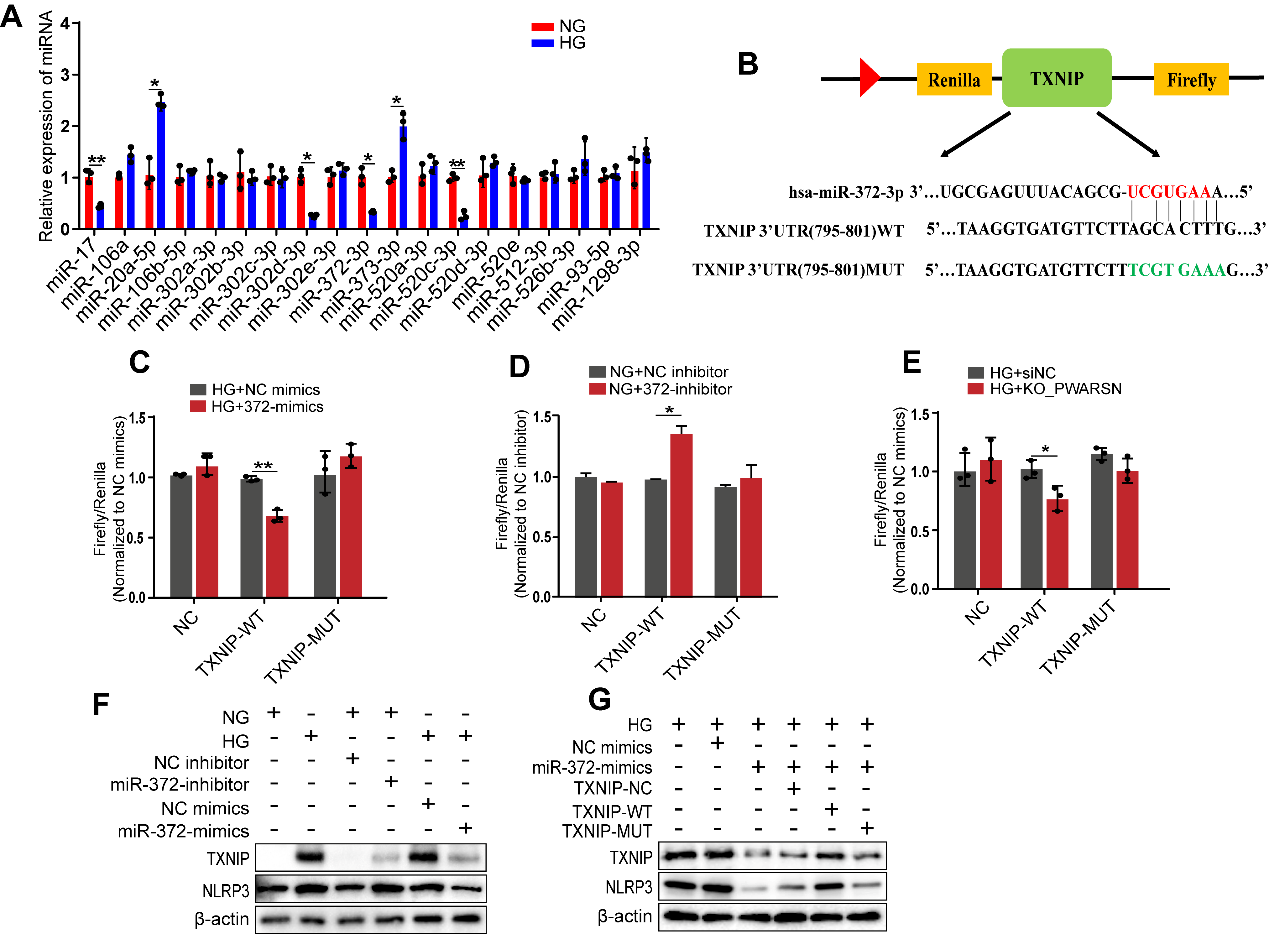


**Figure S7** **MiR-372-3p regulates TXNIP**

**(A)** Relative expression of candidate miRNAs in NG- and HG-treated HK-2 cells. **(B)** Sequence of the wild-type TXNIP 3′-UTR and mutant sequences on the complementary sites of TXNIP 3′-UTR with miR-372-3p. **(C**-**E)** Luciferase activity in HK-2 cells transfected with miR-372-3p mimics **(C)**, miR-372-3p inhibitor **(D)** and in *PWARSN-*knockout HK-2 cells **(E)** luciferase reporters containing empty plasmid, TXNIP and the mutant TXNIP. **(F)** The levels of TXNIP and NLRP3 in HK-2 cells transfected with miR-372-3p mimics and inhibitor. **(G)** Changes in protein levels of TXNIP and NLRP3 in HK-2 cells co-transfected with miR-372-3p mimics and TXNIP plasmid or the mutant plasmid of the 3′-UTR sequence of TXNIP. Data are presented as the means ± SD and significance was determined using unpaired Student’s *t*-test (**A,** **C, D, E**) for two groups. (**P* <0.05; ***P* <0.01; ****P* <0.001; NS: not significant).

**Figure S8**


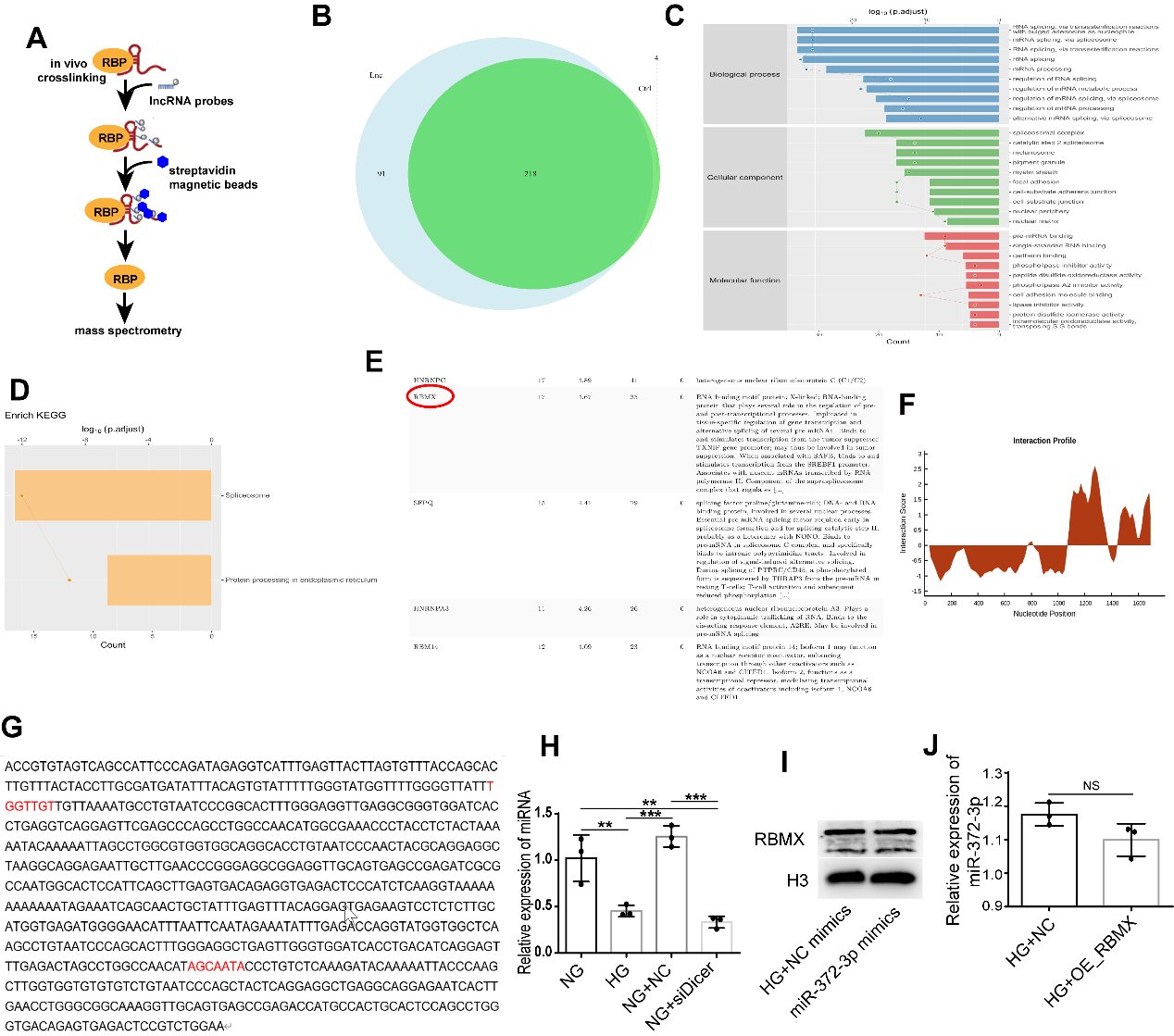


**Figure S8** ***PWARSN* interacts with RBMX**

**(A)** Schematic of the chromatin isolation by RNA purification-mass spectrometry used to identify the endogenous proteins binding to *PWARSN* directly in HK-2 cells. **(B)** 91 proteins interacting with *PWARSN* were identified using mass spectrometry. **(C-D)** GO and KEGG analysis of *PWARSN* binding proteins. **(E)** RBMX was one of the most enriched proteins. **(F)** The interaction sites between *PWARSN* and RBMX as predicted by catRAPID software. **(G)** The binding sites in *PWARSN* sequence that RBMX motifs interacted with *PWARSN*. **(H)** Relative expression of miR-372-3p in NG-treated cells transfected with Dicer-siRNA. **(I)** The effect of miR-372-3p mimics on RBMX by western blotting. **(J)** The effect of RBMX overexpression on miR-372-3p expression by qRT-PCR. Data are presented as the means ± SD, and significance was determined using unpaired Student’s *t*-test (**J**) for two groups and one-way ANOVA (**H**) for multiple groups. (NS: not significant).

**Figure S9**


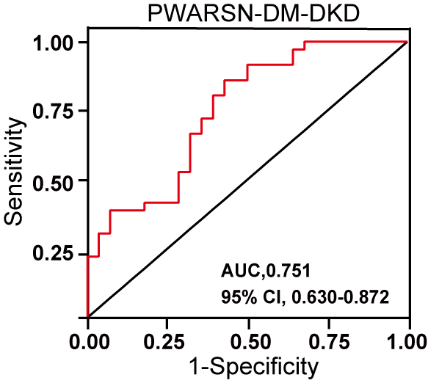


**Figure S9 The ROC analysis of the power of *PWARSN* in urinary sediment samples from patients with DM and DKD**

Receiver operating characteristic curve (ROC) analysis of the power of *PWARSN* in urinary sediment samples from patients with DM and DKD for diagnosing patients with DKD. AUC, area under the curve; CI, confidence interval.

**Supplementary Tables**

**Supplementary Table 1**

**Table S1. Clinical characteristics of patients in pathological samples**

| **Parameters** | **patients with NGT (*n* = 32)** | **DM patients without DKD (*n* = 22)** | **patients with DKD (*n* = 15)** | ***P* value** |
| --- | --- | --- | --- | --- |
| Sex | Male: Female=19:13 | Male: Female=11:11 | Male: Female=10:5 | 0.59 |
| Age (year) | 52.38±8.08 | 55.46±8.52 | 57.00±5.44 | 0.126 |
| Blood glucose (mmol/L) | 4.73±0.57 | 8.67±2.52 | 9.56±2.01 | <0.001^ab^ |
| eGFR (ml/min/1.73m^2^) | 103.83±8.66 | 101.99±12.44 | 61.33±12.04 | <0.001^bc^ |
| Albumin/Urine creatinine ratio (mg/g) | 15.52±8.97 | 15.59±8.66 | 248.47(194.30,265.44) | <0.001^bc^ |
| Duration of disease (year) | N/A | 9.70±0.27 | 10.49±0.17 | 0.66 |

Data are calculated by One-way ANOVA or Kruskal-Wallis Test and presented as mean ± SD or median (25^th^percentile–75^th^ percentile) according to data distribution. Multiple comparison (LSD-t or Bonferroni’s test according to data distribution): a. patients with NGT *vs* DM patients without DKD. b. patients with NGT *vs* patients with DKD. c. DM patients without DKD *vs* patients with DKD. NGT: normal glucose tolerance; DM: diabetes mellitus; DKD: diabetic kidney disease.

| **Supplementary Table 2**  **Table S2.** **Body weight, kidney weight/body weight ratio and biochemical indicators in normal control (NC), diabetic (DM) and saline/Vector/*PWARSN* groups at week 8 after injection** | | | | | | | | | |
| --- | --- | --- | --- | --- | --- | --- | --- | --- | --- |
| **Group** | ***n*** | **BG**  **(mmol/L)** | **BW (g)** | **KI（%）** | **24h-UTP**  **(mg)** | **Scr**  **(μmol/l)** | **BUN**  **(mg/dl)** | **URBP**  **(mg/l)** |  |
| NC | 5 | 6.44±1.32 | 25.15±1.19 | 1.16±0.10 | 2.77（2.17,2.88） | 18.28±1.14 | 25.84±1.58 | 0.08±0.004 |  |
| DM | 5 | 27.44±2.79^a^ | 20.53±0.49^a^ | 1.57（1.38,1.59）^a^ | 15.58±2.37^a^ | 23.84±1.88^a^ | 57.22±6.60^a^ | 0.36±0.31^a^ |  |
| saline | 5 | 6.28±0.66^b^ | 25.56±0.40 ^b^ | 1.28±0.09 | 3.52（3.00,3.57）^b^ | 19.43±0.38 | 29.56±14.34^b^ | 0.15±0.02 |  |
| Vector | 5 | 8.24±0.78^b^ | 25.60±2.15^b^ | 1.14（1.11,1.37） | 3.61±0.36^b^ | 19.59±1.00 | 26.76±2.24^b^ | 0.16±0.04 |  |
| *PWARSN* | 6 | 6.83±1.10^b^ | 24.13±1.10^b^ | 1.15±0.16^b^ | 9.00±1.17^abcd^ | 28.53±3.94^acd^ | 44.81±4.70^acd^ | 0.68±0.09^acd^ |  |
| Data are calculated by One-way ANOVA or Kruskal-Wallis Test and the data are displayed as the means ± SD or median (25^th^percentile-75^th^ percentile). Multiple comparison (LSD-t or Bonferroni’s test according to distribution of data): a. *P*<0.05, *vs* NC；b. *P*<0.05, *vs* DM; c. *P*<0.05, *vs* saline; d. *P*<0.05, *vs* Vector. BG: blood glucose; BW: body weight; KI: kidney weight/body weight ratio; 24h-UTP: 24h urine total protein; Scr: serum creatinine; BUN: blood urea nitrogen; URBP: urinary retinol-binding protein. | | | | | | | | | |

**Supplementary Table 3**

**Table S3. Clinical characteristics of patients in plasma samples**

| **Parameters** | **healthy individuals with NGT (*n* = 56)** | **DM patients without DKD (*n* = 44)** | **patients with DKD (*n* = 53)** | ***P* value** |
| --- | --- | --- | --- | --- |
| Sex | Male: Female=29:27 | Male: Female=21:23 | Male: Female=30:23 | 0.83 |
| Age (year) | 49.77±9.23 | 53.30±10.45 | 57.34±8.75 | <0.001^bc^ |
| Blood glucose (mmol/l) | 4.55(4.23,4.94) | 6.68(5.43,8.74) | 7.67(5.29,10.45) | <0.001^ab^ |
| HbA1c (%) | 5.60(5.30,5.88) | 8.24±1.59 | 8.78±2.23 | <0.001^ab^ |
| Urine protein (g/24h) | 0.08(0.04,0.14) | 0.11(0.08,0.17) | 0.28(0.15,1.06) | <0.001^bc^ |
| eGFR (ml/min/1.73m^2^) | 102.25(94.18, 109.42) | 101.59±14.48 | 90.51(72.04,104.37) | <0.001^bc^ |
| Albumin/Urine creatinine ratio (mg/g) | 6.92±2.26 | 7.40(5.75,9.70) | 102.10(47.35,207.10) | <0.001^bc^ |
| Urine 24h-Microalbumin (mg/l) | 0.08(0.04,0.14) | 0.11(0.08,0.17) | 0.28(0.15,1.06) | <0.001^bc^ |

Data are calculated by One-way ANOVA or Kruskal-Wallis Test and presented as mean ± SD or median (25^th^percentile–75^th^ percentile) according to the distribution of data. Multiple comparison (LSD-t or Bonferroni’s test according to distribution of data): a. healthy individuals with NGT *vs* DM patients without DKD. b. healthy individuals with NGT *vs* patients with DKD. c. DM patients without DKD *vs* patients with DKD. NGT: normal glucose tolerance; DM: diabetes mellitus; DKD: diabetic kidney disease.

**Supplementary Table 4**

**Table S4. Clinical characteristics of patients in urine sediment samples**

| **Parameters** | **healthy individuals with NGT (*n* = 20)** | **DM patients without DKD (*n* = 28)** | **patients with DKD (*n* = 36)** | ***P* value** |
| --- | --- | --- | --- | --- |
| Sex | Male: Female=9:11 | Male: Female=11:17 | Male: Female=17:19 | 0.55 |
| Age (year) | 44.00±13.15 | 54.505.26(46.25,60.50) | 52.75±14.23 | 0.037^ac^ |
| Blood glucose (mmol/l) | 5.26(4.60,5.64) | 6.77(5.34,8.15) | 7.55(5.65,9.91) | 0.01^ab^ |
| HbA1c (%) | 5.45(5.03,5.83) | 8.00(6.93,10.18) | 8.85(7.50,10.53) | <0.001^ab^ |
| Urine protein (g/24h) | 0.06(0.04,0.08) | 0.11(0.08,0.16) | 0.44(0.20,1.50) | <0.001^bc^ |
| eGFR (ml/min/1.73m^2^) | 104.78(101.52, 107.42) | 106.64(99.85,114.26) | 92.73±27.43 | 0.018^bc^ |
| Albumin/Urine creatinine ratio (mg/g) | 7.72±2.86 | 7.50(5.83,8.85) | 95.00(14.33,266.04) | <0.001^bc^ |
| Urine 24h-Microalbumin (mg/l) | 9.74±5.11 | 16.45(12.29,22.71) | 248.63(65.73,1405.40) | <0.001^bc^ |

Data are calculated by One-way ANOVA or Kruskal-Wallis Test and presented as mean ± SD or median (25^th^percentile–75^th^ percentile) according to the distribution of data. Multiple comparison (LSD-t or Bonferroni’s test according to distribution of data): a. healthy individuals with NGT *vs* DM patients without DKD. b. healthy individuals with NGT *vs* patients with DKD. c. DM patients without DKD *vs* patients with DKD. NGT: normal glucose tolerance; DM: diabetes mellitus; DKD: diabetic kidney disease.

**Supplementary Table 5**

**Table S5. siRNA oligos and primers**

**① Primers used in qRT-PCR**

| **Primers** | **Species** | **Sequence** |
| --- | --- | --- |
| PWARSN | Human | F:5’ AGTCAGCCATTCCCAGATAGA 3’  R:5’ AACCCCAAAACCATACCCA 3’ |
| TXNIP | Human | F: 5’ CGCCACACTTACCTTGCCAATG 3’  R: 5’ GCTCTTGCCACGCCATGATG 3’ |
| Txnip | Mouse | F: 5’ CGCCACACTTACCTTGCCAATG 3’  R: 5’ GCTCTTGCCACGCCATGATG 3’ |
| RBMX | Human | F: 5’ ACGAGGACCACCACCACGAAG 3’  R:5’ TCCCAGTCCACTGCTGCTACG 3’ |
| Nlrp3 | Human | F: 5’ GCCACGCTAATGATCGACTTCAATG 3’  R:5’ CCACTCCTCTTCAATGCTGTCTTCC 3’ |
| NLRP3 | Mouse | F: 5’ GCCGTCTACGTCTTCTTCCTTTCC 3’  R:5’ CATCCGCAGCCAGTGAACAGAG 3’ |
| Asc | Mouse | F: 5’ GGACGGAGTGCTGGATGCTTTG 3’  R:5’ CATCTTGTCTTGGCTGGTGGTCTC 3’ |
| miR-372-3p | Human | F:5' cgcgcgAAAGTGCTGCGACATTT 3’  R:5’ gcgCAAAgTgcTTACAgTgc 3’ |
| GAPDH | Human | F:5’GGGAAACTGTGGCGTGAT 3’  R:5’GAGTGGGTGTCGCTGTTGA 3’ |
| β-actin | Human | F:5’CCTGGCACCCAGCACAAT 3’  R:5’GGGCCGGACTCGTCATAC 3’ |
| β-actin | Mouse | F:5’ GTGCTATGTTGCTCTAGACTTCG 3’  R:5’ATGCCACAGGATTCCATACC 3’ |
| U6 | Human | F: 5’CTCGCTTCGGCAGCACA 3’  R: 5’AACGCTTCACGAATTTGCGT 3’ |

**② Probes used in FISH assays**

| **Genes** | **Sequence** |
| --- | --- |
| *PWARSN* | CTGCCTTAGCCTCCTGCGTAGTTG |
| miR-372-3p | ACGCTCAAATGTCGCAGCACTTT |

**③ SiRNAs used to silence the targets**

| **Targets** | **Sequence** |
| --- | --- |
| siPWARSN-1 | CAGTGGCATTAACATAACTC |
| siPWARSN-2 | CCTTGCGATGATATTTACAG |
| siPWARSN-3 | GAGTTCAGTGGCATTAACA |
| ASO PWARSN-1 | ACTGTGCCAGGTACTAATTT |
| ASO PWARSN-2 | GGACTACAGTCGCGTGTCA |
| ASO PWARSN-3 | TCAGAATCTAGGCCTATCT |
| siRNA RBMX-1 | AAGTTCTCGTGATACTAGA |
| siRNA RBMX-2 | CTGTATCACGTGGAAGAGA |
| siRNA RBMX-3 | GACTATCCATCAAGAGGAT |
| siRNA-Dicer | CGAAGGCAGUGCUACCCAAdTdT |

**④ Primers used in ChIP-qPCR assays**

| **Primers** | **Species** | **Sequence** |
| --- | --- | --- |
| ***PWARSN*** | Human | F:5'AGTCAGCCATTCCCAGATAGA3’  R:5’ AACCCCAAAACCATACCCA3’ |
| **TXNIP** | Human | F: 5’ CGCCACACTTACCTTGCCAATG 3’  R: 5’ GCTCTTGCCACGCCATGATG 3’ |

**Supplementary Table 6**

**Table S6. The list of lncRNAs in lncRNA-TXNIP co-expression network**

| **GeneSymbol** | **lncRNA** | **PCC** | **PCC_group** |
| --- | --- | --- | --- |
| TXNIP | NR_037911 | 0.902104 | + |
| TXNIP | T337986 | 0.966884 | + |
| TXNIP | ENST00000606587 | -0.93826 | - |
| TXNIP | ENST00000439819 | 0.922731 | + |
| TXNIP | NR_027158 | 0.936524 | + |
| TXNIP | T181493 | 0.905212 | + |
| TXNIP | T018085 | 0.941567 | + |
| TXNIP | T019592 | 0.90127 | + |
| TXNIP | T249006 | 0.908382 | + |
| TXNIP | TCONS_00017204 | 0.919951 | + |
| TXNIP | T251871 | 0.95776 | + |
| TXNIP | T356261 | 0.939376 | + |
| TXNIP | NR_003952 | -0.94364 | - |
| TXNIP | uc021tyb.2 | -0.91777 | - |
| TXNIP | ENST00000366321 | -0.9264 | - |
| TXNIP | ENST00000454832 | -0.92438 | - |
| TXNIP | ENST00000454380 | 0.910708 | + |
| TXNIP | T213262 | 0.942209 | + |
| TXNIP | ENST00000440971 | -0.92082 | - |
| TXNIP | NR_027345 | 0.94379 | + |
| TXNIP | T017205 | -0.9255 | - |
| TXNIP | ENST00000467198 | 0.901272 | + |
| TXNIP | ENST00000608290 | 0.948271 | + |
| TXNIP | NR_047498 | 0.905866 | + |
| TXNIP | TCONS_00021786 | 0.917374 | + |
| TXNIP | TCONS_12_00021062 | 0.944168 | + |
| TXNIP | ENST00000416502 | 0.927014 | + |
| TXNIP | NR_022011 | 0.91287 | + |
| TXNIP | GSE61474_TCONS_00087239 | -0.93232 | - |
| TXNIP | TCONS_00028716 | 0.915897 | + |
| TXNIP | T326109 | 0.973903 | + |
| TXNIP | NR_027159 | 0.964677 | + |
| TXNIP | ENST00000569214 | -0.91711 | - |
| TXNIP | ENST00000535921 | 0.917663 | + |
| TXNIP | ENST00000422807 | 0.957882 | + |
| TXNIP | T280226 | -0.92489 | - |
| TXNIP | NR_110801 | 0.966021 | + |
| TXNIP | TCONS_00008434 | 0.908073 | + |
| TXNIP | ENST00000519013 | 0.903423 | + |
| TXNIP | ENST00000455011 | 0.950684 | + |
| TXNIP | NR_033557 | -0.91758 | - |
| TXNIP | NR_110008 | -0.92655 | - |
| TXNIP | uc.17+ | 0.913923 | + |
| TXNIP | ciRS-7 | 0.923733 | + |
| TXNIP | T143531 | -0.90098 | - |
| TXNIP | T027085 | 0.974095 | + |
| TXNIP | NR_027160 | 0.940765 | + |
| TXNIP | GSE61474_TCONS_00220963 | 0.963116 | + |
| TXNIP | T099030 | 0.978311 | + |
| TXNIP | NR_040092 | 0.916336 | + |
| TXNIP | T120295 | 0.908438 | + |
| TXNIP | T038110 | 0.910503 | + |
| TXNIP | NR_045196 | 0.979012 | + |
| TXNIP | ENST00000552780 | 0.905381 | + |
| TXNIP | TCONS_00014969 | 0.921553 | + |
| TXNIP | T035585 | 0.928661 | + |
| TXNIP | NR_040054 | -0.90112 | - |
